# Exploring the Mechanisms and Therapeutic Approaches of Mitochondrial Dysfunction in Alzheimer’s Disease: An Educational Literature Review

**DOI:** 10.1007/s12035-024-04468-y

**Published:** 2024-09-10

**Authors:** Mostafa Hossam El Din Moawad, Ibrahim Serag, Ibraheem M. Alkhawaldeh, Abdallah Abbas, Abdulrahman Sharaf, Sumaya Alsalah, Mohammed Ahmed Sadeq, Mahmoud Mohamed Mohamed Shalaby, Mahmoud Tarek Hefnawy, Mohamed Abouzid, Mostafa Meshref

**Affiliations:** 1 Faculty of Pharmacy, Clinical Department, Alexandria Main University Hospital, Alexandria, Egypt; 2 Faculty of Medicine, Suez Canal University, Ismailia, Egypt; 3 Faculty of Medicine, Mansoura University, Mansoura, Egypt; 4 Faculty of Medicine, Mutah University, Al-Karak, Jordan; 5 Faculty of Medicine, Al-Azhar University, Damietta, Egypt; 6 Department of Clinical Pharmacy, Salmaniya Medical Complex, Government Hospital, Manama, Bahrain; 7 Ministry of Health, Primary Care, Governmental Health Centers, Manama, Bahrain; 8 Misr University for Science and Technology, 6th of October City, Egypt; 9 Faculty of Medicine, Ain Shams University, Cairo, Egypt; 10 Faculty of Medicine, Zagazig University, Zagazig, Egypt; 11 Department of Physical Pharmacy and Pharmacokinetics, Faculty of Pharmacy, Poznan University of Medical Sciences, Rokietnicka 3 St., 60-806 Poznan, Poland; 12 Doctoral School, Poznan University of Medical Sciences, 60-812 Poznan, Poland; 13 Department of Neurology, Faculty of Medicine, Al-Azhar University, Cairo, Egypt

**Keywords:** Mitochondrial Dysfunction, Alzheimer’s Disease, Therapeutic Modalities

## Abstract

Alzheimer’s disease (AD) presents a significant challenge to global health. It is characterized by progressive cognitive deterioration and increased rates of morbidity and mortality among older adults. Among the various pathophysiologies of AD, mitochondrial dysfunction, encompassing conditions such as increased reactive oxygen production, dysregulated calcium homeostasis, and impaired mitochondrial dynamics, plays a pivotal role. This review comprehensively investigates the mechanisms of mitochondrial dysfunction in AD, focusing on aspects such as glucose metabolism impairment, mitochondrial bioenergetics, calcium signaling, protein tau and amyloid-beta-associated synapse dysfunction, mitophagy, aging, inflammation, mitochondrial DNA, mitochondria-localized microRNAs, genetics, hormones, and the electron transport chain and Krebs cycle. While lecanemab is the only FDA-approved medication to treat AD, we explore various therapeutic modalities for mitigating mitochondrial dysfunction in AD, including antioxidant drugs, antidiabetic agents, acetylcholinesterase inhibitors (FDA-approved to manage symptoms), nutritional supplements, natural products, phenylpropanoids, vaccines, exercise, and other potential treatments.

## Introduction

Alzheimer’s disease (AD) is the leading cause of dementia and presents a substantial challenge to healthcare systems worldwide [1, 2]. It is distinguished by a gradual deterioration in cognitive function, leading to impairment in daily activities and a rise in morbidity and mortality among older people [1, 2]. FDA-approved AD medications encompass both symptom management and disease treatment. Symptom management drugs include brexpiprazole, donepezil, galantamine, memantine, a combination of memantine and donepezil, and rivastigmine [3]. For disease treatment, lecanemab, a disease-modifying immunotherapy, is used. It treats mild cognitive impairment or mild AD by removing abnormal beta-amyloid to help reduce the number of plaques in the brain [3, 4].

**Fig. 1:**
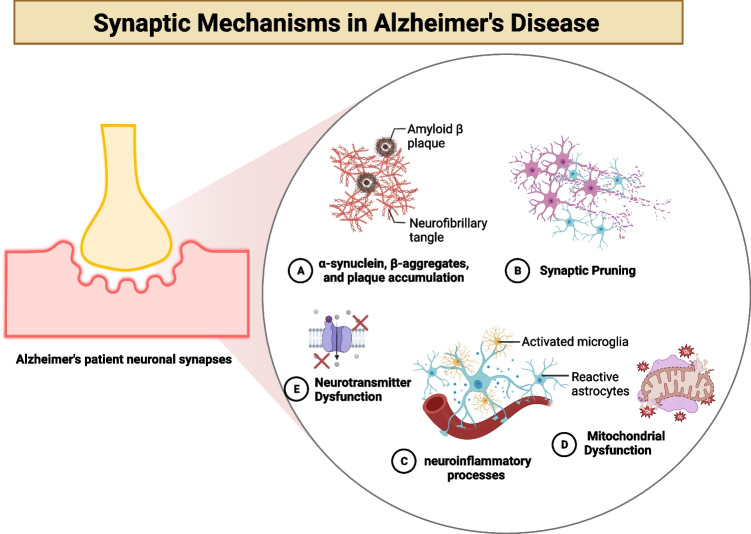
Synaptic mechanisms in Alzheimer’s disease (AD). **A** AD is characterized by the accumulation of tau protein tangles and amyloid beta (Aβ) plaques in the brain, disrupting synapses’ normal functioning. The regular operation of synapses is compromised due to the interference of oligomers with neurotransmitter action. Microtubules, which are essential for maintaining the structure and function of synapses, are adversely affected by tau protein tangles that disrupt their typical structure and function [14]; **B** synaptic pruning is a process through which the brain eliminates redundant or underused synapses. This process is essential for the normal functioning of the brain. However, it may be implicated in AD. In AD, synaptic pruning is excessively activated, leading to a reduction in functional synapses. Consequently, the brain ends up with fewer synapses, which could contribute to the cognitive decline associated with AD [15, 16]; **C** microglia, the brain’s immune cells, can become activated due to persistent inflammation. While microglia are necessary for removing Aβ plaques, they may also contribute to synaptic dysfunction. The pro-inflammatory cytokines secreted by activated microglia can impair synaptic function. Additionally, the activation of astrocytes, another type of brain cell, can release inflammatory substances that exacerbate synaptic dysfunction [17]; **D** the onset of AD has been linked to mitochondrial dysfunction. When mitochondria fail, they produce reactive oxygen compounds that can disrupt proteins, lipids, and DNA. This oxidative stress may play a role in the loss of functional connections, a characteristic feature of AD [13], and (**E**) neurotransmitters are chemical messengers essential for transmitting signals between neurons. AD is characterized by decreased neurotransmitters, such as acetylcholine, which plays a crucial role in memory and learning. The dysfunction of synapses caused by this neurotransmitter deficiency may contribute to the cognitive decline associated with AD [18]

The pathophysiology of AD is complex and involves multiple factors [5–12]. Mitochondria, essential for cellular energy metabolism, can impair neuronal function when dysfunctional [8, 13]. Synapses, the connections between neurons, are critical for communication and signal transmission in the brain. Several mechanisms contribute to synapse dysfunction in AD, including amyloid beta peptide (Aβ) and tau protein [14], synaptic pruning [15, 16], inflammatory processes [17], mitochondrial dysfunction, and cholinergic signaling, particularly acetylcholinesterase [18–21] (Fig. 1).

Research suggests that mitochondrial dysfunction plays a central role in the progression of AD [22, 23]. This literature review aims to highlight and provide up-to-date information on the mechanism of mitochondrial dysfunction and the therapeutic modalities for mitigating mitochondrial dysfunction in AD.

## Mechanism of Mitochondrial Dysfunction in Alzheimer’s Disease

### Glucose Metabolism Impairment and AD

Despite accounting for only 2% of body weight, the brain consumes 25% of the body’s oxygen and 25% of its glucose. These demonstrated how vulnerable our brains are to energy metabolism abnormalities, to the point that a minor change in energy metabolism is significantly associated with a disturbance in the functioning of the nervous system. Impaired energy metabolism is one of the early and most persistent symptoms of AD [24]. The primary necessary substrate for the adult human brain and its cerebral endothelial cells is glucose [25]. A 55-kDa isoform of glucose transporter 1 (GLUT1) imports glucose into cerebral endothelial cells [26]. After that, glucose travels through glycolysis, followed by the pentose-phosphate route, lactate fermentation, or mitochondrial metabolism [25].

A growing amount of data points to decreased glucose consumption as an early and persistent characteristic of AD, occurring up to decades before the disease’s onset [27–30]. When comparing AD brains (especially the hippocampus and cortex) to individuals without dementia, fluoro-2 deoxyglucose positron-emission tomography (FDG-PET) was used to discover a greater decline in glucose consumption. Furthermore, in the early stages of AD, the posterior cingulate cortex was shown to be the most metabolically damaged of all brain areas [27]. Moreover, people with moderate cognitive decline, a prodromal phase of AD, show glucose hypometabolism but to a lower extent in terms of quantity or geographic distribution. This suggests low glucose metabolism affected the disease’s onset [29]. Apolipoprotein E (ApoE ε4) allele is recognized as a risk factor for AD and moderate cognitive decline. Indeed, it is frequently mentioned as the primary genetic factor of AD [31–36]. In their 84-month longitudinal FDG PET investigation, Paranjpe et al. [31] showed that patients with moderate cognitive decline had an ApoE ε4-associated brain region-specific glucose metabolism pattern. Decades before dementia may manifest, in their 20 s, young persons with the ApoE ε4 gene were found to have glucose hypometabolism in the brain regions that are susceptible to it [37].

Furthermore, in patients with AD, the amount and geography of glucose underutilization reflected the distribution of diminished synaptic function and density in distinct brain areas, coinciding with the severity of symptoms [38, 39]. These days, cerebral glucose hypometabolism is recognized as a characteristic of the illness, and measuring it with FDG-PET is turning it into a biomarker for early AD identification and entirely accurate and sensitive moderate cognitive decline to AD conversion prediction [40, 41].

The relationship between amyloid plaque formation and glucose low metabolism has been examined using amyloid PET biomarkers and FDG PET. Longitudinal A depositions (the predominant type of amyloid) were found in practically every cortical area in carriers of autosomal-dominate AD mutations 15–25 years before the expected age of beginning, which appeared before glucose hypometabolism in specific cortical regions approximately 5–10 years later. In these circumstances, glucose underutilization may arise due to A depositions in AD development [30, 42, 43]. Diminished local glucose consumption was linked to worldwide amyloidosis. Comparing the same patients revealed weak correlations between regional amyloid pathology and regional glucose hypometabolism (just one location out of 404 showed a negative correlation between glucose metabolism and amyloid plaque deposition) [44]. These findings might imply that glucose underutilization is vital in defining the clinical manifestations of the illness, even if it happens incidentally in autosomal-dominate AD carriers. Given this, as well as the recurrent failures of A-centered clinical studies, one may argue that it is too late to target A in AD or even those with moderate cognitive decline after years of amyloid pathology launching deadly cascades of events. Impaired energy metabolism, on the other hand, may afford a wider window for therapeutic intervention [24].

### Mitochondrial Bioenergetics in AD

Several studies have identified abnormalities in mitochondrial-related metabolic processes associated with AD through gene expression analyses, providing compelling evidence of dysfunctional mitochondrial bioenergetics in patients with AD [45–49]. Liang et al. [46] conducted a genome-wide transcriptome study using postmortem brains of patients with AD and controls from various brain regions, focusing on the activity of 80 metabolically relevant nuclear genes in non-tangle-bearing neurons obtained through laser-capture microdissection. Their findings revealed a significant decrease in the expression of nuclear genes encoding components of the mitochondrial electron transport chain in patients with AD’s posterior cingulate cortex, hippocampus CA1, and middle temporal gyrus, with reductions of 70%, 65%, and 61%, respectively. In contrast, the visual cortex exhibited only a 16% decrease in expression, indicating relative protection from metabolic deficits in aging and AD [47, 48].

Another study utilized postmortem human hippocampus tissues to analyze the expression of mRNA transcripts involved in glucose metabolism in patients with AD, revealing substantial downregulation of 15 out of 51 members associated with pathways related to oxidative phosphorylation (OXPHOS), glycolysis, and the TCA cycle [49]. Mastroeni et al. [45] investigated hippocampal specimens from healthy controls, individuals with amnestic mild cognitive impairment, and AD cases, confirming a significant reduction in OXPHOS genes in AD, particularly those expressed by the nucleus. Interestingly, individuals with mild cognitive impairment exhibited higher levels of these genes compared to both patients with AD and healthy controls.

### Mitochondria and Calcium Signaling

**Fig. 2:**
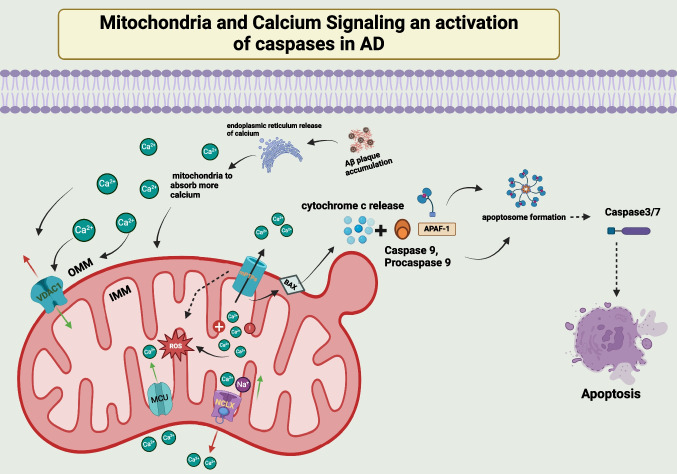
Illustration of calcium ions (Ca2 +) signaling in mitochondrial dysfunction-associated neuronal apoptosis in Alzheimer’s disease (AD). The buildup of Aβ in cortical neurons is associated with releasing calcium from the endoplasmic reticulum, leading to increased cytosolic calcium ion levels and enhanced mitochondrial calcium absorption. Mitochondria and the endoplasmic reticulum play a crucial role in maintaining calcium homeostasis by transferring calcium ions out of the mitochondria and into the matrix via various channels and transporters [voltage-dependent anion-selective channel 1 (VDAC1); the Na + -dependent mitochondrial calcium efflux transporter (NCLX), and the mitochondrion calcium uniporter (MCU)]. Overloading mitochondria with calcium ions triggers the opening of mitochondrial transition pores (mPTPs), releasing cytochrome c, activating caspase activation, and initiating apoptosis [50–54, 59]

The active transport of calcium ions (Ca^2+^), triggered by the action potential, is essential for neuronal development and function [50]. It functions as a messenger, activating the calcium channel to transfer depolarization calcium ions to the neuron’s presynaptic end. This releases neurotransmitters via exocytosis, which gives the postsynaptic neuron the action potential [51, 52]. The presynaptic zone has an increase in calcium concentrations due to this mechanism [51]. Calcium homeostasis is one of the most critical functions performed by mitochondria and the endoplasmic reticulum [53, 54]. It reduces calcium concentrations by transferring calcium ions out of the mitochondria and into the matrix via the voltage-dependent anion-selective channel 1 (VDAC1) on the outer membrane, the Na + -dependent mitochondrial calcium efflux transporter (NCLX), and the mitochondrion calcium uniporter (MCU) on the inner membrane of the mitochondria [55–58]. When the mitochondria are overloaded with calcium ions, the inner membrane’s permeability of mitochondria transition pores (mPTPs) opens, releasing cytochrome c from the cells and triggering caspases in the cytoplasm, triggering apoptosis [59] (Fig. 2). Aβ plaque accumulation in synaptic mitochondria plays a significant role in calcium dyshomeostasis in AD [59].

Furthermore, it is hypothesized that the accumulation of Aβ in cortical neurons instigates calcium release from the endoplasmic reticulum. This event elevates the levels of cytosolic calcium ions, thereby prompting mitochondria to absorb more calcium [60, 61]. The subsequent rupture of the mitochondrial membrane can be attributed to the high calcium concentrations within the mitochondria. This phenomenon can be elucidated by activating pro-apoptotic proteins, opening mPTPs, and augmentation in ROS [59]. Notably, this dysregulation of calcium at the mitochondrial level has been observed in the brains of patients diagnosed with AD [62, 63].

### Aβ, Protein Tau, and Associated Synapse Dysfunction

An accumulation has been associated with synaptic dysfunction and neurotoxicity. It obstructs anterograde mitochondrial transport to the synapses, neurotransmitter release, and synaptic vehicle renewal [64–68]. Furthermore, it was demonstrated that Aβ promoted and inhibited long-term depression and N-metylo-D-asparaginowy (NMDA)-dependent long-term potentiation in synaptic connections [69, 70]. In a similar vein, tau has been linked to synaptic impairment in patients with AD. Through its interaction with Synaptogyrin-3, it was discovered to limit synaptic vesicles’ mobility and diminish neurotransmitters’ release from vesicles [64, 71, 72]. Furthermore, tau has been linked to reduced mitochondrial axonal transport movement by interfering with microtubules, which in turn interferes with dynein and kinesin binding, diminishing neurotransmission [73, 74]. Interestingly, increased synaptic activity has been linked to increased tau diffusion to synapses, exacerbating synaptic dysfunction [75]. It was discovered that interactions between dynamin-related protein 1 (Drp1) and a rise in hyperphosphorylated tau mitochondrial fission, which in turn reduces the amount of functional mitochondria present in the synapse [11]. Memory impairment and cognitive impairment caused by AD are triggered by slower and disrupted neurotransmission as a result of progressive synaptic dysfunction. Individuals with AD experience dementia, and the condition proceeds as a result of synaptic degradation and subsequent neuronal death in their brains [76].

### Mitophagy and Autophagy

Mitophagy is a particular type of autophagy through which mitochondria are attacked and degraded. These cellular processes play a crucial role in energy conservation, cellular destruction, and preventing the accumulation of damaged organic molecules [77]. Many studies revealed that mitophagy processes are deformed in AD [78–81].

Most studies report the Pink–Parkin mitophagy pathway; however, cardiolipin-induced mitophagy has been reported in mouse models with AD [82]. Mitophagy markers increase with the disease progression, as reported in postmortem brain tissue and animal models. Yet, the cytosolic Parkin concentration is decreased, reducing its availability for mitophagy [83]. The cause of the accumulated mitochondria that are targeted for mitophagy is unclear. However, some studies reported that cells with presenilin-1 (PSEN1) mutations [84] or cells expressing the apoE4 gene [85] exhibit lysosomal dysfunction.

It is unknown what is generating the increased recruiting of Parkin to mitochondria; it might be due to mitochondrial membrane potential depolarization, which is produced by amyloid interlinkage with mitochondria. Furthermore, amyloid contributes to ROS generation, signaling mitophagy’s start by boosting Parkin accumulation [77]. However, studies using animal and cell models have demonstrated that tau can either boost the recruitment of Parkin to mitochondria [79] or prevent its movement from the cytoplasm [80, 81].

The preparedness of a mitochondrion for mitophagy can be influenced by several factors, including the formation of ROS and its breakdown of mitochondrial membrane potential. The permeability of the mPTP is a transmembrane protein found in the inner mitochondrial layer that is critical in determining the degree of cellular death and mitophagy [86]. It has been reported that the mPTP function may be disturbed in AD, as a study showed a further constant activation of the pore in cells compared to healthy controls [87].

### Aging

Because of accumulating damage and limited self-repair, old age is a substantial contributory factor for many neurodegenerative illnesses. As we age, our mitochondria’s shape and function alter substantially. Several studies, for example, found age-related changes in the structure of mitochondrial membranes, including the loss of cristae and inner membrane vesicles. Apoptogens are released into the cytoplasm because of the outer membrane breach caused by the division of adenosine triphosphate (ATP) synthase dimers into monomers. Furthermore, vesiculations of the membrane’s inner layer and the breaking of ATP synthase dimers cause a considerable decrease in ATP [88].

According to research, age-related synaptic mitochondria aggregation disrupts synaptic activities such as ATP synthesis and calcium equilibrium, which are required for efficient depolarization-evoked neurotransmitter vesicle formation and plasticity. As a result, cognitive function and memory are impaired. Nonsynaptic mitochondria are less sensitive to age-dependent alterations and the accumulation of A aggregates [89, 90].

Aging is the leading risk factor for the beginning of sporadic AD; prevalence increases with age, from 2% in those 65–69 to 25% in those 90 + [91]. Numerous cohort studies indicate that age must be considered when evaluating AD treatments’ safety and possible efficacy [92]. The accumulation of free radicals may accelerate aging in addition to metabolic decline.

Oxidative damage to mitochondrial macromolecules, especially mtDNA, would be most severe as mitochondria are the cell’s primary source of free radical production [93]. Reduced activity of antioxidant enzymes such as glutathione reductase, catalase, superoxide dismutase, and glutathione peroxidase is also associated with chronic free radical accumulation in the AD brain [94, 95].

Moreover, reports indicate that a decline in proteasome activity brought on by aging may facilitate the deposition of Aβ and tau [96, 97]. Consequently, these aging-related mechanisms establish an endless loop that leads to advanced mitochondrial dysfunction as well as the buildup of Aβ and tau, the two main pathogenic characteristics of AD.

### Inflammation

Pathogen-associated molecular patterns (PAMPs) originate from pathogens or exogenous ligands, while damage-associated molecular patterns (DAMPs) are endogenously produced molecules released into the extracellular environment following tissue damage. Pattern recognition receptors identify PAMPs and DAMPs, subsequently triggering intracellular signal transduction pathways that enhance innate immune responses. Due to the similarities between mitochondria and bacteria, when mitochondrial material escapes into the cytosol or extracellular environment, it activates pattern recognition receptors signaling by serving as a PAMP or DAMP [98]. As a result, mitochondria control the signals that cause inflammation.

DAMPs and PAMPs in the central nervous system induce pro-inflammatory immune responses in glial cells, resulting in chronic neuroinflammation and speeding up the etiology of neurodegenerative diseases such as AD [99, 100]. There is evidence that mtDNA causes in vivo neuroinflammation, as when mtDNA or mitochondrial lysates are injected into the hippocampus dentate gyri, pro-inflammatory signaling is triggered [101].

The introduction of mitochondria or mtDNA into the hippocampus area phosphorylates NF-B, increases TNF mRNA synthesis, and lowers myeloid cells 2 (TREM2) expression, all of which are markers of AD pathogenesis [102, 103] and are included in phagocytic and anti-inflammatory pathways [104, 105]. Notably, mitochondrial lysates likewise increase endogenous APP and Aβ [101].

### Mitochondrial DNA (mtDNA)

mtDNA is susceptible to oxidative damage due to its proximity to generating ROS, the absence of protective histones, and limited repair mechanisms [106]. In the brains of patients with AD, mtDNA exhibits approximately ten times more oxidized bases and three times more oxidative damage than nuclear DNA, potentially leading to mutations impairing mitochondrial function, cell death, and disease progression [107]. Mutations in mtDNA have been associated with cognitive impairments and are implicated in the onset of AD [106]. Specific maternally inherited genetic changes, known as mtDNA single nucleotide polymorphisms and haplogroups, have been linked to an increased risk of AD [108–110]. Notably, mtDNA accumulates mutations during aging, the primary risk factor for AD [111]. Furthermore, alterations in mtDNA, such as elevated 5-methylcytosine levels in the D-loop region in AD pathology brain samples with and reduced D-loop region methylation in peripheral blood mtDNA from patients with late-onset AD, can impact mtDNA transcription and function [112, 113].

ROS or the autophagic/lysosomal system may release mtDNA, initiating or exacerbating AD development by triggering a pro-inflammatory response. While this phenomenon has been observed in other conditions, such as cardiomyopathy and systemic inflammation, the specific mechanisms underlying the effects of released mtDNA in AD remain unclear and require additional investigation [114].

### Mitochondria-Localized microRNAs (mitomiRs)

The pathogenesis of AD has been linked to mitochondrial miRNAs, which play a crucial role in regulating mitochondrial function. Dysfunctional miRNAs in neurons, often due to oxidative stress, can lead to increased production of ROS by mitochondria [115]. Specific mitochondrial miRNAs, such as miR-98 and miR-15b, have been shown to support redox balance, while miR-204 and miR-34a have been found to elevate ROS generation and impede the activity of antioxidant enzymes [116–119]. Dysregulation of these miRNAs can lead to neuronal death due to heightened oxidative stress in AD, while reduced levels of miR-98 and miR-15b can increase ROS production and oxidative damage. The transmission of synaptic information and plasticity heavily relies on mitochondrial function. Specific mitochondrial miRNAs, including miR-484, miR-132, and miR-212, have been demonstrated to enhance neurotransmission [120, 121].

**Fig. 3:**
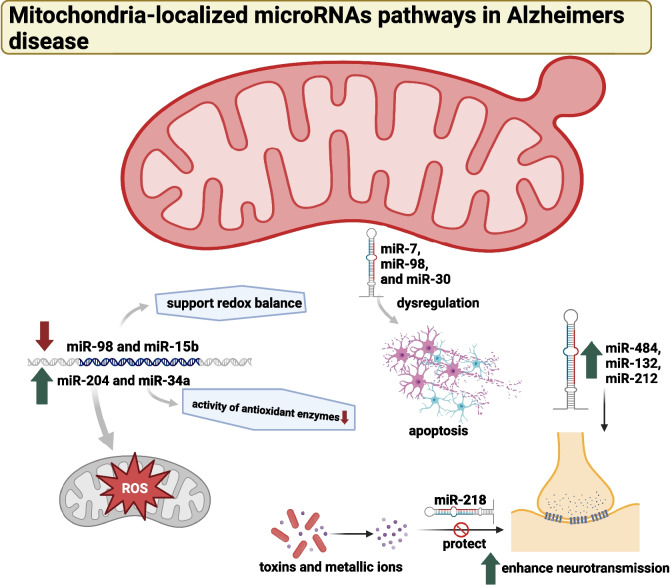
The role of mitochondrial miRNAs in the pathogenesis of Alzheimer’s disease (AD). Dysregulation of specific miRNAs, often due to oxidative stress, can lead to increased production of ROS and neuronal death. Specific miRNAs, such as miR-98 and miR-15b, support redox balance, while others, like miR-204 and miR-34a, elevate ROS generation. The figure also highlights the role of miRNAs in synaptic information transmission and plasticity, with miR-484, miR-132, and miR-212 enhancing neurotransmission. Dysregulation of these miRNAs can contribute to synaptic dysfunction in AD. The figure further depicts the role of miRNAs in apoptosis, a mechanism regulating neuronal survival and death. Dysregulation of miRNAs implicated in apoptosis, such as miR-7, miR-98, and miR-30, can lead to increased apoptosis and neuronal death, disrupting learning and memory pathways [115–125]

Additionally, miR-218 has been identified as playing a role in protecting neurons from toxins and metallic ions that can induce synaptic toxicity [122]. The dysregulation of miRNAs involved in synaptic plasticity, such as miR-132 and miR-484, is likely to contribute to the observed synaptic dysfunction in AD [117, 121]. Programmed cell death, or apoptosis, is a fundamental mechanism for regulating the survival and death of neurons, particularly in the context of AD. Dysregulation of mitochondrial miRNAs implicated in apoptosis, such as miR-7, miR-98, and miR-30, has been observed, potentially leading to increased apoptosis and neuronal death [118, 123, 124]. Extensive neuronal death disrupts pathways associated with learning and memory, further exacerbating the cognitive deficits seen in AD [125]. Therefore, the dysregulation of mitochondrial miRNAs in AD will likely contribute to various aspects of the condition, including oxidative damage, synaptic dysfunction, and neuronal death. Overall, research on mitochondrial miRNAs and their role in neurodegenerative diseases holds promise for developing novel diagnostic and therapeutic approaches for AD and other neurodegenerative disorders (Fig. 3).

### Genetics

Genetic variations in mitochondrial regulatory pathways can lead to a gradual decline, ultimately resulting in compromised mitochondrial integrity and mtDNA damage, leading to mtDNA alteration dysfunction and disease [126]. Genetics can influence mitochondrial dysfunction and increase the risk of developing AD through various mechanisms. Aberrations in genes responsible for encoding mitochondrial proteins can disrupt mitochondrial function, resulting in the accumulation of oxidative damage and a decrease in energy production. Some of these mutations are associated with the production and metabolism of Aβ, which are known to aggregate in the brains of individuals with AD. Early-onset, autosomal dominant familial AD has been linked to mutations in the amyloid precursor protein (APP), PSEN1, and PSEN2 genes, typically manifesting in the fifth or sixth decade of life [126]. However, exceptions exist, and generally, if an individual develops AD after the age of 60 and does not have a parent who was affected by the disease before the age of 60, genetic testing is unlikely to reveal an autosomal dominant mutation in the APP, PS1, or PS2 genes. Individuals who develop sporadic AD at a younger age are thought to have a higher genetic predisposition for the disease. The presence of the APOE4 allele is frequently observed in these patients, indicating that APOE4 may be a risk factor for the early onset of AD in individuals carrying this allele [127].

### Hormones

Several studies have indicated sex-specific differences in mitochondrial dysfunction in the brain and that age-related declines in sex hormone levels may play a role in such dysfunction due to the critical regulatory role of hormones in mitochondrial activity [128]. Moreover, research has shown that ovulation significantly reduces mitochondrial respiration, suggesting that female sex hormones like progesterone and estrogen have a more pronounced impact on mitochondrial activity than testosterone [129]. Estradiol, the primary estrogen in humans, has been found to enhance OXPHOS activity, reduce the generation of ROS, and preserve mitochondrial membrane potential [130]. A postmenopausal mouse model investigation revealed that cognitive decline associated with estrogen deficiency coincides with abnormal mitochondrial biogenesis, disrupted mitochondrial dynamics, reduced mitophagy, and mitochondrial dysfunction [131]. Similarly, progesterone has been shown to decrease oxidative stress and increase mitochondrial energy production [132]. Additionally, studies have suggested that testosterone deficiency may potentially impair brain substantia nigra mitochondria by increasing oxidative stress and reducing the activity of complex I, underscoring the potential influence of testosterone on mitochondrial dysfunction in the brain [133]. Furthermore, it has been proposed that the age-related decline in sexual steroid production could contribute to the deterioration of brain mitochondria [128].

### Electron Transport Chain and Krebs Cycle

Numerous studies have highlighted alterations in the electron transport chain (ETC) and tricarboxylic acid (TCA) cycle, the two paramount metabolic pathways within mitochondria. Researchers have reported a decrease of 30–40% in the activity of complex IV [134–137] and alpha-ketoglutarate dehydrogenase (aKGDH) [138–140], both crucial components of these metabolic pathways. Recent studies on human donor livers have provided evidence that the activity of the mitochondrial respiratory chain (complexes I, II, III, IV) and Krebs cycle enzymes (aconitase, citrate synthase) does not significantly differ before and after a 4-h preservation period across all study groups (*p* > 0.05) [141]. Interestingly, low-risk livers that were clinically viable (*n* = 8) exhibited lower activities of complexes II–III following 4-h perfusion compared to high-risk livers (73 nmol/mg/min vs. 113 nmol/mg/min, *p* = 0.01). Applying actively oxygenated and air-equilibrated end-ischemic hypothermic machine perfusion (HMP) did not induce oxidative damage to aconitase, and the integrity of the respiratory chain complexes was maintained. This suggests that mitochondria likely adapt their respiratory function in response to varying oxygen levels in the perfusate during end-ischemic HMP. Given these findings, the activities of complexes II–III warrant further investigation as potential biomarkers for viability [141].

A more exhaustive screening of the activities of TCA cycle enzymes in AD [142] revealed a heterogeneous response: some enzymes exhibited decreased activity (e.g., pyruvate dehydrogenase, alpha-ketoglutarate dehydrogenase, isocitrate dehydrogenase), others showed increased activity (e.g., succinate dehydrogenase and malate dehydrogenase), while the activity of the remaining four enzymes remained unchanged (e.g., aconitase). These alterations are presumed to result in a decline in succinyl-CoA, an intermediate of the TCA cycle produced by alpha-ketoglutarate dehydrogenase and utilized in the subsequent reactions catalyzed by succinate dehydrogenase and malate dehydrogenase. Succinyl-CoA serves as a precursor for heme synthesis [143, 144]; thus, a decrease in succinyl-CoA levels would be expected to lead to a decline in heme production [145, 146].

## Therapeutic Modalities for Mitigating Mitochondrial Dysfunction in Alzheimer’s Disease

**Fig. 4:**
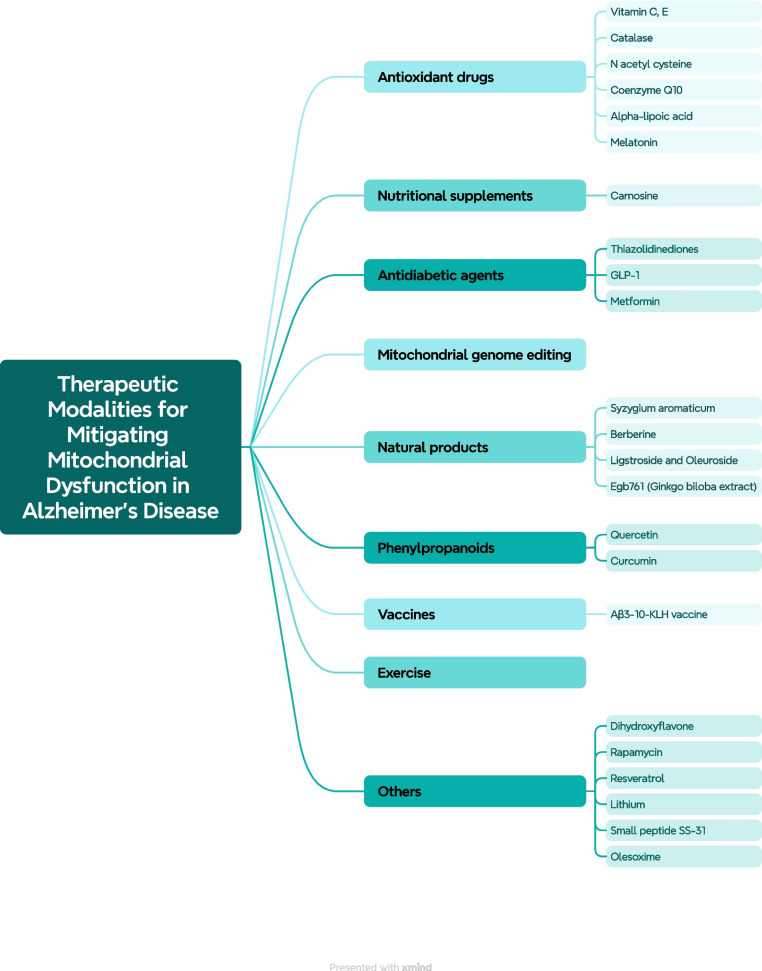
Summary of therapeutic modalities for mitigating mitochondrial dysfunction in Alzheimer’s disease. Preclinical models (catalase, N-acetylcysteine, Coenzyme Q10, melatonin, exenatide, metformin, carnosine, clove, berberine, ligstroside, and oleuroside, Egb761, quercetin, dihydroxyflavone, nilotinib, rapamycin, resveratrol, Aβ3-10-KLH vaccine and olesoxime), and clinical models (vitamin C and E, alfa-lipoic acid, thiazolidinediones, curcumin, lithium and small peptide SS-31)

Numerous studies have connected mitochondrial dysfunction to the etiology of AD, involving oxidative stress, faulty electron transport chain, mtDNA damage, and improper mitochondrial dysfunction (Fig. 4). The following section highlights potential therapeutics for AD in preclinical (catalase, N-acetylcysteine, Coenzyme Q10, melatonin, exenatide, metformin, carnosine, clove, berberine, ligstroside and oleuroside, Egb761, quercetin, dihydroxyflavone, nilotinib, rapamycin, resveratrol, Aβ3-10-KLH vaccine, and olesoxime), and clinical models (vitamin C and E, alfa-lipoic acid, thiazolidinediones, curcumin, lithium, and small peptide SS-31). Table 1 summarizes the mechanisms of proposed therapeutic modalities [106–126, 147–153].

**Table 1:** Summary of the proposed agents targeting mitochondrial dysfunction in Alzheimer’s disease

Treatment	Proposed mechanism	Ref
Antioxidant agents		
Vitamins C and E	Scavenges ROS and reduces lipid peroxidation	[106, 107]
Catalase*	Decreased BACE activity, lessened aberrant APP processing, decreased oligomeric Aβ buildup, and defense against oxidative damage	[108]
*N*-acetylcysteine*	Influencing the levels of energy- and mitochondria-related proteins	[109]
Coenzyme Q10*	CoQ10 reduces oxidative stress and amyloid pathology and improves behavioral performance	[110]
*alfa-lipoic acid*	Improve mitochondria function by enhancing different respiratory complexes and ATP levels	[111]
Melatonin*	Improve mitophagy, restore mitochondrial function, attenuate Aβ pathology	[112]
Mitochondria genome editing*		
Using mitochondria-specific mitoCas9 and the clustered regularly interspaced short palindromic repeats (CRISPR) method		[147]
Antidiabetic agents		
Thiazolidinediones	Induces mitochondrial biogenesis, improves memory, lowers Aβ burden, and lowers phospho‐τ	[113]
GLP1 (exenatide)*	Enhancing OXPHOS activity, reducing oxidative stress, and improving mitochondrial biogenesis	[114]
Metformin*	Mitophagy is induced by activating SIRT1, AMPK, and Parkin and suppressing the activities of complex 1 and mTOR	[115, 118, 119]
Nutritional supplements		
Carnosine*	Prevent intracellular Zn2 + dyshomeostasis and intraneuronal Aβ deposition	[116]
Natural products		
Syzygium aromaticum*	Anti-oxidative capacity and can scavenge ROS, activate SIRT1, and downregulate γ-secretase level	[117]
Berberine*	Modulate mitochondrial bioenergetics and glutathione metabolism pathways	[120]
Ligstroside and oleuroside*	Restore brain ATP level and enhance the capacity of respiratory chain complexes	[149]
Egb761 (*Ginkgo biloba* extract)*	Suppress the JNK signaling pathway	[150]
Phenylpropanoids		
Quercetin*	Increase in mitochondrial biogenesis and reduction in free radicals	[121]
Curcumin	Modulates mitochondria stress response, a potent antioxidant, and regulates mitochondria redox balance	[122]
Others		
Dihydroflavone*	Reducing oxidative stress, mitochondrial dysfunction, and insulin resistance	[124]
Nilotinib*	Increases the clearance of amyloid beta (Aβ) by promoting mitophagy mediated by Parkin	[125]
Rapamycin*	Alleviates AD-like behaviors and synaptic plasticity deficits in APP/PS1 mice by correcting mitophagy	[126]
Resveratrol*	- The impact of Aβ on mitochondrial oxidative stress, neuroprotection, and antiaging - Elevation of COX levels autophagic and mitophagies’ stimulation	[123]
Aβ3-10-KLH vaccine*	Induce a high level of anti-Aβ antibodies	[151]
Lithium	Selective inhibitory effect on GSK-3β	[152]
Small peptide SS-31	Inhibits oxidative stress and restores normal mitochondrial function	[153]
Olesoxime*	Enhancing the activity of respiratory chain complexes and reversing complex IV activity	[148]

^*^Preclinical models

### Antioxidant Drugs

#### Vitamin C, E

**Fig. 5:**
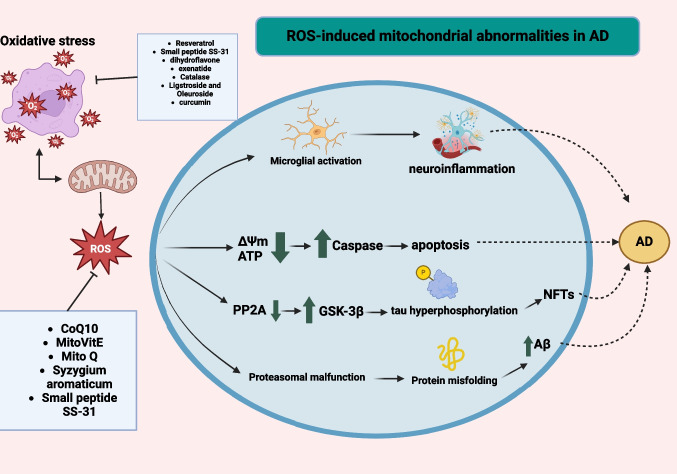
Reactive oxygen species (ROS)-induced mitochondrial abnormalities in Alzheimer’s disease (AD). The overproduction of ROS or an impaired antioxidant system can shift the cellular redox balance towards oxidative imbalance. ROS, generated during cellular respiration, can harm mitochondria and neuronal function. An increase in ROS can lead to a reduction in mitochondrial membrane potential (ΔΨm) and ATP generation, negatively impacting mitochondrial energy stores, disrupting energy metabolism, and compromising dynamics and mitophagy. Furthermore, ROS can increase caspase activity, initiating apoptosis. Overproduction of ROS can also inhibit phosphatase 2A (PP2A), which activates glycogen synthase kinase (GSK) 3β, leading to tau hyperphosphorylation and the accumulation of neurofibrillary tangles [157]

Exogenous antioxidants, such as vitamins C and E, which can reduce ROS-induced damage, are one strategy to enhance mitochondrial function and halt disease development (Fig. 5). Unfortunately, clinical trials have not proved these antioxidants’ usefulness since they cannot localize into mitochondria or cross the blood–brain barrier. Researchers added a novel group of naturally occurring antioxidants termed triphenylphosphonium to the mix to circumvent this barrier. This lipophilic cation boosts the efficiency of antioxidants in restoring mitochondrial health due to its capacity to localize to the negatively charged mitochondrial membrane. One example is MitoVitE, a vitamin E molecule connected to a triphenylphosphonium cation. This enables its rapid uptake into mitochondria, and it has been found to reduce mitochondrial damage induced by oxidative stress and protect against loss of mitochondrial membrane potential in rats [154]. On the other hand, individuals with AD who participated in a 1-year open clinical study received daily supplements containing 1000 mg of vitamin C and 400 IU of vitamin E. Antioxidant vitamins in cerebral fluid increased due to the treatment. However, the progression of AD was not significantly affected [155]. Furthermore, a meta-analysis of vitamin C, E, and carotene levels found that in patients with AD, vitamin E levels were considerably lower than in the control group; however, little difference was seen for vitamin C or carotene [156]. These findings suggest that increasing the intake of vitamin E–rich foods may be beneficial in preventing AD.

#### Catalase

Catalase is an enzyme that aids in breaking hydrogen peroxide, a poisonous byproduct of cellular metabolism linked to mitochondrial malfunction and AD pathology. A study discovered that the mitochondria-targeted antioxidant catalase can prevent aberrant APP processing, lower A levels, and increase A-degrading enzymes in AD mice, showing its promise as a treatment strategy [158].

#### N-acetyl cysteine

N-acetyl cysteine is the primary source of glutathione, an antioxidant vital in avoiding oxidative stress and mitochondrial dysfunction. According to research on an AD animal model, N-acetyl cysteine treatment improved Aβ-induced abnormalities in mitochondria and synaptic degeneration, reduced oxidative stress, and increased mitochondrial function [159]. However, more investigation is needed to determine the ideal dosage and length of N-acetyl cysteine therapy to address mitochondrial dysfunction in patients with AD.

#### Coenzyme Q10 (CoQ10)

AD and other neurodegenerative illnesses may benefit from Coenzyme Q10 (CoQ10) as a treatment [160]. Although CoQ10 may have neuroprotective properties, research on in vitro and animal models has shown conflicting findings in AD clinical trials [160]. Patients with AD exhibited equivalent serum/plasma CoQ10 levels to controls, according to a comprehensive review and meta-analysis of studies evaluating tissue CoQ10 levels in patients with dementia and controls [161]. Human investigations have produced conflicting outcomes, although CoQ10 has demonstrated significant neuroprotective effects in laboratory models of AD and other dementias [161].

#### Alpha-lipoic Acid

Alpha-lipoic acid has been shown to have various beneficial effects on pathogenic pathways of dementia, including reducing oxidative stress, inflammation, and mitochondrial dysfunction [162]. A study investigated the impact of alpha-lipoic acid treatment (600 mg/day) on cognitive performance in patients with AD with and without diabetes mellitus and found that alpha-lipoic acid therapy may be effective in slowing cognitive decline in patients with AD with insulin resistance [163]. Furthermore, a review article suggests that alpha-lipoic acid may have potential therapeutic benefits in preventing several diseases, including AD, due to its antioxidant and anti-inflammatory properties [164].

#### Melatonin

Melatonin’s antioxidant properties and sleep–wake cycle modulation are only two of its numerous roles. Melatonin is widely known for protecting against aging, neurological ailments, and mitochondrial diseases. However, its effect on mitophagy in AD is unknown. An experiment on an AD-prone mouse model indicated that oral melatonin treatment increased mitophagy, restored mitochondrial function, decreased A pathology, and improved cognitive performance, hinting that it might be used as a therapeutic alternative for managing AD [165].

### Antidiabetic Agents

#### Thiazolidinediones

Thiazolidinediones are a family of insulin-sensitizing drugs that have been identified to have potential therapeutic benefits in treating AD due to their unique agonists of the gamma receptor for peroxisome proliferator (PPAR). They have also been proposed as innovative and potentially effective treatments for neurodegenerative illnesses. In preclinical studies, rosiglitazone treatment had positive effects. Rodent studies show that rosiglitazone reduces the quantity of phosphorylated tau protein, improves cognition, boosts mitochondria biogenesis, and lowers A burden [166]. Furthermore, in a Phase 2 human study, rosiglitazone-treated patients with AD (ApoE 4 non-carriers) displayed enhanced cognitive performance [167]. Advantages were not shown in later phase 3 trials [168].

#### GLP-1

GLP-1 agonists have been licensed to heal type 2 diabetes, including exenatide. It has also been postulated that these agents may have neuroprotective effects due to their impact on mitochondrial activity [169]. GLP-1 analogs have been shown to improve mitochondrial function by increasing OXPHOS activity, decreasing oxidative stress, increasing glucose uptake and utilization, and boosting mitochondrial biogenesis. Exenatide, a GLP-1 receptor agonist, has shown promise in lowering mitochondrial dysfunction and cognitive decline in 5xFAD transgenic mice, implying that it might one day be utilized to prevent mitochondrial damage in AD [170]. Furthermore, the research looked at the impact of subcutaneous liraglutide (25 nmol/kg/qd for 8 weeks) in 5 FAD mice and A-treated astrocytes. Liraglutide was discovered to increase neuronal support, reduce neuronal death, and alleviate mitochondrial dysfunction in the brain by activating the cyclic adenosine 3′,5′-monophosphate (cAMP)/phosphorylate protein kinase A (PKA) pathway. Furthermore, GLP-1 reduced mitochondrial fragmentation in A-treated astrocytes, enhanced mitochondrial failure, ROS excessive production, mitochondrial membrane potential collapse, and cell toxicity [171].

#### Metformin

Metformin, a treatment for type 2 diabetes mellitus, has demonstrated potential in managing conditions such as AD [172]. Clinical studies have indicated that metformin is associated with enhanced cognitive function and a reduced risk of developing AD; however, these effects may be influenced by variables such as APOE-ε4 status and diabetes status [172]. Mechanistic investigations have revealed the impact of metformin on AD etiology and pathophysiology, encompassing neuronal loss, neural dysfunction, tau phosphorylation, Aβ deposition, chronic neuroinflammation, insulin resistance, altered glucose metabolism, and mitochondrial dysfunction [172]. Recent research suggests that metformin prevents mitochondrial-mediated apoptosis and diminishes the generation of ROS in mitochondrial respiratory-chain complex 1 [173]. Metformin has been shown to delay aging and mitigate the progression of aging-related diseases, including AD, by targeting critical aging-related events, such as mitochondrial dysfunction [174]. Furthermore, metformin activates SIRT1, AMPK, and Parkin while inhibiting complex 1 and mTOR activities, thereby inducing mitophagy [175]. Additionally, promising results of metformin have been observed in disease models, including increased lifespan in mice, reduced hyperphosphorylated τ in a diabetes mouse model, and reversal of AD features in APP/PS1 [176].

### Acetylcholinesterase Inhibitors/NMDA-Receptor Antagonist

The only pharmacological treatments approved for AD are acetylcholinesterase inhibitors (ChEIs) and the NMDA receptor antagonist memantine [177]. Despite their seemingly modest benefits [178, 179], a substantial body of evidence supports their efficacy in enhancing cognition and cost-effectiveness [180–190]. One of the earliest pathological findings in AD is the degeneration of basal forebrain cholinergic neurons, which precedes the onset of dementia [180, 190]. The progression of AD correlates more closely with dysfunction in the cholinergic system than with the amyloid plaque load [191]. Furthermore, a reduction in the volume of the basal forebrain precedes changes in the volume of the hippocampus and predicts the cortical spread of AD pathology [192].

ChEIs function by maximizing the availability of endogenous acetylcholine in the brain [193]. However, few randomized clinical trials have investigated the efficacy of ChEIs in AD following 1 year of treatment [194–198] or have conducted patient follow-ups beyond this point [197]. Studies examining long-term cognitive decline are complicated due to high attrition rates and loss of follow-up [197]. Some follow-up studies of cohorts treated with ChEIs for Alzheimer’s dementia have demonstrated minor cognitive benefits at 2, 3, and over 10 years [199–201]. A positive short-term response to ChEIs can also delay admission to nursing homes [202].

Xu et al. [203] emphasized that ChEIs are associated with modest cognitive benefits that persist over time and with a reduced risk of mortality, which could be partially attributed to their cognitive effects. Among all ChEIs, only galantamine has demonstrated a significant reduction in the risk of progressing to severe dementia. Other studies have reported associations between the use of ChEIs and a decreased risk of myocardial infarction, stroke, and death in patients with AD [204–207].

### Nutritional Supplements

AD is known by zinc (Zn^2+^) dyshomeostasis with the pathological accumulation of Aβ and tau protein in the brain [208]. A study investigated the potential impact of carnosine, a dipeptide, on zinc (Zn^2+^) chelation and AD-like cognitive deficits in 3xTg-AD mice [209]. The findings demonstrated that carnosine effectively chelates intracellular Zn^2+^ and reduces intraneuronal Aβ deposition in the hippocampus. However, it was ineffective in addressing tau pathology in the brain [208]. The administration of carnosine at a concentration of 20 mM during acute and intense Zn^2+^ rises allowed for examining its chelating properties [208]. The supplementation of carnosine exhibited a favorable trend towards improved cognitive performance in 3xTg-AD mice, as evidenced by the reduced latency to locate the platform [208]. The study suggests that carnosine may serve as a potential dietary supplement for mitigating intracellular Zn^2+^ dyshomeostasis and intraneuronal Aβ deposition, which are significant contributors to the onset and progression of AD [208].

### Natural Products

#### *Syzygium aromaticum* (Clove)

Shekhar et al. [210] investigated the impact of *Syzygium aromaticum* (or clove) on sirtuin (SIRT1) and the oxidative balance in the context of Aβ-induced toxicity to determine whether clove could modulate the oxidative pathway. The findings revealed that clove exhibits anti-oxidative properties, which can scavenge ROS, activate SIRT1, and downregulate secretase levels [210]. These results suggest that clove may offer a holistic approach to treating neurodegenerative diseases, potentially leading to the development of innovative therapeutics for AD. Given its high oxygen radical absorbance capacity value and ability to balance Vata while stimulating nerves, clove may also serve as a potential anti-aging agent [211].

#### Berberine

Chinese medicinal herbs, such as berberine, have a long history of use in treating various illnesses, including AD [212]. berberine has been associated with numerous neuroprotective benefits that may enhance the brain’s energy state in the early stages of AD [213]. A recent study revealed that berberine mitigates abnormalities in crucial energy and glutathione metabolism pathways in AD cells and modulates mitochondrial bioenergetics, slowing basal respiration and reducing the production of pro-inflammatory cytokines from activated microglial cells [214]. These findings suggest that berberine may benefit from disrupted metabolic pathways in the early stages of AD development [214]. Additionally, the study investigated the synergistic effects of berberine and pioglitazone, a PPAR agonist. It indicated that both drugs may have comparable potential benefits for AD, as they bind to the PPAR protein with similar affinities [214].

#### Ligstroside and Oleuroside

Two secoiridoids, ligstroside and oleuroside, are bioactive chemicals in olive oil [215], which may play an essential role in preventing AD due to their capacity to increase mitochondrial activity [216]. Grewal et al. studied the effects of two metabolites and ten distinct pure phenolic secoiridoids at deficient concentrations on mitochondrial activity in early AD cellular model SH-SY5Y-APP695 cells [149]. The studied secoiridoids markedly raised these cells’ baseline ATP levels. The compounds that significantly impacted ATP levels were ligstroside, oleacein, oleeuroside, and oleocanthal. They were also tested for their effects on mitochondrial respiration. The only substances that may increase the respiratory chain complexes’ capability were ligstroside and oleocanthal [149].

To investigate the underlying molecular mechanisms of these activities, qRT-PCR was utilized to assess the expression of genes associated with respiration, anti-oxidative ability, and mitochondrial biogenesis. Only ligstroside increased mRNA expression of complex I, GPx1, SIRT1, and CREB1 [149]. Additionally, oleocanthal, not ligstroside, reduced A 1–40 levels in SH-SY5Y-APP695 cells. To assess the in vivo effects of pure secoiridoid, the two most promising compounds, oleocanthal, and ligstroside, were tested in an aging mouse model [217]. Female NMRI mice were fed a diet supplemented with 50 mg/kg of ligstroside or oleocanthal for 6 months. Compared to aged control animals, mice administered with ligstroside exhibited significantly prolonged lifespan, improved spatial working memory, and restored brain ATP levels [149]. These findings indicate that pure ligstroside significantly enhances mitochondrial bioenergetics in early AD and brain aging models through pathways that may not affect A production. Furthermore, ligstroside enhances cognitive function and extends the lifespan of aged mice [149]. Therefore, ligstroside holds promise as a potential therapeutic agent for the prevention and treatment of AD.

#### Egb761 (*Ginkgo* biloba Extract)

Flavonoids and terpenoids are among the bioactive substances found in EGb761, a standardized extract made from *Ginkgo biloba* leaves [218]. The possible therapeutic effects of EGb761 on brain function have been assessed in clinical studies; its impact on age-related dementias and AD has received particular attention [219].

In recent work, researchers used an in vitro cell culture model and an in vivo AD rat model to evaluate the regulation of A-induced necroptosis by EGb761 and associated roles in AD pathogenesis [150]. They showed that EGb761 may suppress the JNK signaling pathway in vitro and in vivo. This could explain why it may avoid A-induced tissue morphogenesis, cell death, and necroptosis in BV2 cells and enhance cognitive performance. These findings support the potential therapeutic effects of plant extracts like Egb761 in treating neurodegenerative illnesses like Alzheimer’s [150].

Randomized double-blind trials were carried out in the study, requiring a minimum of 22 weeks of treatment for EGb761 at a dose of 240 mg/day and 12 weeks for ChEIs or memantine [220]. The study assessed how Medicare enrollees with dementia or moderate cognitive impairment were managed clinically with amyloid PET imaging. This multisite longitudinal trial, called the Imaging Dementia-Evidence for Amyloid Scanning (IDEAS) study, aimed to determine whether amyloid PET imaging was associated with changes in clinical care after that [220, 221].

### Phenylpropanoids

Phenylpropanoids are a class of natural chemicals found in plants with a wide range of biological actions [222]. Because of their anti-inflammatory, antioxidant, and neuroprotective qualities, they have been examined for their potential therapeutic implications in mitochondrial dysfunction associated with AD. Among the phenylpropanoids, quercetin and curcumin have been widely studied for their potential advantages in treating mitochondrial dysfunction in AD [223].

#### Quercetin

Quercetin, a naturally occurring flavonoid, has been demonstrated to have protective benefits in animal models of AD. It is being studied for its efficacy in treating mitochondrial dysfunction in AD [224]. Studies on the effect of quercetin on mitochondrial function have yielded promising results. Quercetin treatment boosted mitochondrial biogenesis, decreasing free radicals in neuronal SH-SY5Y cells [225]. Chronic oral quercetin therapy decreased -amyloidosis and tauopathy in a triple transgenic AD mouse model, leading to cognitive functional recovery [226]. A meta-analysis of 14 research found that quercetin had neuroprotective benefits in multiple AD models, including the potential to ameliorate mitochondrial abnormalities [224]. Moreover, a study exploring the effects of quercetin liposomes administered nasally demonstrated improved cognitive behavior and reduced oxidative stress markers in the hippocampus of an AD animal model [227].

#### Curcumin

Curcumin, a natural chemical found in turmeric, has received interest for its possible neuroprotective and cognitive-enhancing qualities in treating or preventing neurodegenerative illnesses such as AD [228, 229]. Several studies have investigated curcumin’s potential in addressing mitochondrial dysfunction in AD. Notably, one study demonstrated curcumin’s ability to suppress Aβ-induced oxidative damage, improve memory impairment, and enhance microglial labeling near Aβ [229]. Additionally, a review article explored curcumin’s effects on cognition and proposed strategies to overcome current limitations and improve its efficacy [230]. Studies in vitro and in vivo have shown that curcumin can decrease Aβ production, inhibit Aβ aggregation, and promote Aβ clearance [231]. Its mechanism of action involves attenuating amyloid precursor protein maturation, suppressing beta-secretase 1 expression, and binding to Aβ peptides to prevent aggregation [232].

Additionally, curcumin activates the Wnt/β-catenin and PERK/eIF2/ATF4 pathways, leading to BACE-1 inhibition and accelerated Aβ clearance [233]. Moreover, a study discussed using curcumin nanoformulations as theranostic agents to optimize its pharmacokinetic properties alongside other bioactive compounds [234]. However, a systematic review evaluating the efficacy of curcumin in patients with AD, encompassing dosages ranging from 100 mg to 4 gm/day, indicated inconsistent results likely attributed to limitations such as small sample sizes and short study durations, underscoring the necessity for further research in this field [228].

### Vaccines

The Aβ3-10-KLH vaccine has been developed as a potential treatment for AD by stimulating an immune response against Aβ [235]. This vaccine includes Aβ3-10, a fragment of the protein believed to be highly immunogenic. In a study on a mouse model of AD [151], the A3-10-KLH vaccination induced a high level of anti-A antibodies in mice, improving cognitive and learning abilities. The vaccination reduced A plaques and oligomers in the cortex and hippocampus of mice, which are areas of the brain most affected by AD [151]. Additionally, the vaccination inhibited neuron loss and apoptosis, which are significant pathogenic factors in AD. Moreover, the immunization increased the levels of Preps, a protein that may degrade A in brain mitochondria. Consequently, the A3-10-KLH vaccination shows promise as a therapy for AD, potentially enhancing cognitive performance while reducing pathogenic markers associated with the condition [151].

### Exercise

It has been proposed that physical activity may help enhance cognitive abilities among people with AD. According to a recent investigation conducted on APP/PS1 transgenic mice [236], high-intensity interval training (HIIT) and moderate-intensity continuous training (MICT) workouts were found to increase memory and exploratory behavior. In the Morris water maze test [237], both workouts increased navigation and swimming distance, with no significant difference between the two activities [236]. In the spatial probe test, both workouts enhanced the frequency of platform crossings and the percentage of platform quadrant distance, improving memory capacity. Furthermore, both workouts enhanced exploratory behavior in the open field test, as indicated by the number of probing, total time in the center region, and total distance in the central area. Body weight did not differ considerably across groups; however, the HIIT and MICT groups increased their exercise capacity significantly. These data demonstrate that independent of body weight, HIIT and MICT may improve cognitive performance in patients with AD [236].

### Others

#### Dihydroxyflavone

In a study using a rat model, it was observed that 7,8-dihydroxyflavone (7,8-DHF), a naturally occurring flavonoid present in certain plants, enhanced cognitive function and decreased neurodegeneration by mitigating oxidative stress, mitochondrial dysfunction, and insulin resistance. These findings suggest that 7,8-DHF holds promise as a potential therapeutic intervention for AD in humans. Additionally, the study demonstrated that 7,8-DHF restored cognitive impairment in a rat AD model by addressing oxidative imbalance and dysfunction of mitochondrial enzymes [238].

#### Rapamycin

Rapamycin, a pharmacological agent, has emerged as a promising intervention for enhancing healthy aging and longevity in animals. Its potential use in treating mitochondrial disorders in AD has also been investigated. According to a study, rapamycin therapy increased mitochondrial activity and reduced oxidative stress in a mouse model of AD [239]. Another study revealed that even in the absence of detectable improvements in mitochondrial dysfunction, low-dose oral rapamycin was sufficient to prolong the lifespan of a mouse model with authentic mtDNA disease resulting from a mutation in the thymidine kinase 2 (TK2) [240].

#### Resveratrol

Grapes, apples, blueberries, plums, and peanuts produce natural non-flavonoid polyphenol resveratrol. It exists naturally as a phytoalexin [241]. Numerous bioactivities of resveratrol have been demonstrated, such as anti-aging, anti-inflammatory, cardiovascular protection, anti-cancer, anti-diabetes mellitus, anti-obesity, and neuroprotective properties [242]. In rats, resveratrol at doses of 20 and 40 mg/kg/day was beneficial in lowering the expression of pro-inflammatory markers and mitigating the memory and learning deficits caused by Aβ [243]. Rats with vascular dementia showed enhanced learning and memory when resveratrol (25 mg/kg) was given intragastrically daily. Moreover, it raised glutathione levels, superoxide dismutase activity, and malondialdehyde levels in vascular dementia-affected rats' hippocampal and cerebral cortex [244].

#### Lithium

Lithium, a treatment for psychiatric illnesses, has been found to have the potential to treat neurodegenerative disorders, including AD, due to its neuroprotective and neurotrophic properties [245]. GSK-3β, a kinase protein implicated in multiple physiological processes related to neurodegeneration, is selectively inhibited by lithium. Its inhibitory action on GSK-3β has been demonstrated to lessen Aβ generation, stop tau phosphorylation, and make it easier to induce long-term potentiation in AD-affected mice [152]. Human studies have also shown a substantial positive correlation between long-term lithium medication and a lower incidence of dementia in elderly bipolar illness patients [246]. Additionally, long-term subtherapeutic lithium medication has raised levels of brain-derived neurotrophic factor, lowered AD-related CSF fluid biomarkers [247], and slowed the deterioration in cognitive and functional abilities in patients with amnestic mild cognitive impairment [248]. The neuroprotective mechanisms of lithium may also be related to its regulation of energy metabolism and mitochondrial efficiency, including the activation of the Wnt signaling pathway [249].

#### Small Peptide SS-31

SS-31, a mitochondrial peptide, has shown promise as a possible therapy for AD [250]. The peptide belongs to the Szeto-Schiller family of tiny cell-permeable peptides and binds to the inner mitochondrial membrane without requiring mitochondrial membrane potential or energy [251]. Elamipretide, MTP-131, and Bendavia are the brand names for these drugs. SS-31 is more effective than standard antioxidants like vitamin E [153]. It inhibits oxidative stress and restores normal mitochondrial function. Mitochondria oversee glucose homeostasis, and mitochondrial failure is linked to oxidative stress and malfunction. These have been connected to the development of metabolic and neurological diseases. The usage of MT-targeted drugs like SS-31 can aid in the reduction of oxidative stress and mitochondrial damage [251]. SS-31 has been found to have neuroprotective effects by protecting the synapses, reducing Aβ accumulation, and preventing mitochondrial dysfunction [250]. The peptide has shown promise in preclinical studies for treating AD, indicating its potential as a novel therapeutic agent.

#### Olesoxime

Olesoxime (TRO19622) is a medication tested in AD animal models to see how it affects mitochondrial dysfunction and amyloid precursor protein processing [252]. TRO19622 was given to 3-month-old mice with AD-like features and wild-type mice for 15 weeks in research [148]. In dissociated brain cells and brain tissue homogenates, the drug’s effects on mitochondrial membrane potential, adenosine triphosphate levels, respiration, citrate synthase activity, Aβ levels, and malondialdehyde levels were studied. The results showed that TRO19622 alleviated mitochondrial dysfunction by increasing respiratory chain complex activity and reversing complex IV activity and mitochondrial membrane potential [148]. Furthermore, the medication protected dissociated brain cells against complex I activity inhibition. TRO19622, on the other hand, was shown to enhance the levels of A1-40 in the brains of mice and HEKsw cells. This study reveals that while TRO19622 may have mitochondrial advantages, the increased production of A1-40 may have negative consequences. More studies are needed to determine TRO19622’s potential as a feasible therapy for AD [148].

## Future Research Directions and Potential Roadblocks

The development of therapeutic drugs that specifically target mitochondrial dysfunction is an increasing focus in AD management. An ideal drug may enhance mitochondrial function, reduce oxidative stress, and prevent neuronal death [157, 253]. We have highlighted the latest scientific progress toward developing such medications in Table 2. However, the development of formulations targeting mitochondria faces explicitly numerous challenges.

**Table 2:** Latest proposed mitochondrial drugs in the treatment of Alzheimer’s disease

NCT	Status	Interventions	Enrollment	Location
NCT03514875	Withdrawn	MitoQ	12	United States
NCT02142777	Completed	S -Equol (AUS-131)	15	United States
NCT04842552	Recruiting	Hydralazine hydrochloride	424	Iran
NCT02711683	Completed	DL-3-n-butylphthalide	92	China
NCT00675623	Completed	Dimebon	598	United States
NCT00951834	Completed	Epigallocatechin-gallate	21	Germany
NCT03999879	Recruiting	Measure of OxPhos upregulation	45	United States
NCT04740580	Recruiting	Glycine, N-acetylcysteine, alanine	52	United States
NCT04044131	Completed	Metabolic cofactor, sorbitol	120	Turkey
NCT00678431	Completed	Resveratrol with glucose, and malate	27	United States
NCT04701957	Recruiting	Ketogenic diet	70	France
NCT04018092	Recruiting	Active NIR-PBM, Sham NIR-PBM	168	United States
NCT04430517	Recruiting	Nicotinamide riboside	50	United States
NCT05040048	Completed	Clinical, biological, and imaging assessment	220	France, Germany, Spain, Sweden
NCT02062099	Completed	[18F]DPA-714 PET/ [18F]AV-45 PET/neuropsychological assessment	25	France
NCT03860792	Recruiting	Ketogenic diet, therapeutic lifestyles changes diet	80	United States
NCT05040321	Recruiting	MIB-626	50	United States
NCT03859245	Unknown	Ketogenic diet, photobiomodulation	30	United States
NCT05383833	Recruiting	Creatine monohydrate	20	United States
NCT05591027	Recruiting	Centella asiatica product	48	United States
NCT03702816	Enrolling by invitation	GE180 PET scan	70	United States
NCT00829374	Completed	Dimebon	1003	United States
NCT05343611	Recruiting	Combination of high protein diet and physical exercise protocol, HPP Choko, HPP/VE Choko	75	Italy
NCT01388478	Completed	R-pramipexole	20	United States
NCT02460783	Completed	Boost (R) 5–2 diet, healthy living diet	129	United States
NCT05617508	Recruiting	Nicotinamide riboside 1000 mg daily in total, nicotinamide riboside dose escalation (up to 3000 mg daily in total)	80	Norway
NCT03101085	Completed	S-equol	40	United States
NCT01498263	Completed	Not provided	682	United States

First, the precise mechanisms of action of these drugs are still not fully understood, and further research is needed to elucidate these mechanisms and optimize the efficacy of these drugs, particularly those with promising results in preclinical models such as CoQ10 [161], melatonin [165], olesoxime [148], SS-31 [250, 251], lithium [152, 245, 248, 249], vaccines [151, 235], berberine, and pioglitazone [212–214].

Second, there is difficulty in diagnosing the extent of mitochondrial functioning and dysfunction and determining the drug dose required to produce the desired modulation in mitochondrial functioning [77, 254, 255]. Available AD diagnosis includes cognitive testing, imaging of Aβ and tau pathology in various brain parts, and cerebrospinal fluid assays [256]. However, these techniques have disadvantages, including limited availability, high cost, and invasive procedures employed with their results and integrity under question. Technological advancement has led to researchers speculating novel biomarkers involved in the disorder’s pathogenesis, such as 5-methylcytosine levels in patients with late-onset AD [112, 113], complexes II–III in ETC, and Krebs cycle [141] and mtDNA [106–113]. Developing mitochondrial biomarkers could be an excellent approach, as mitochondrial functions are standard in various cell types and present in sporadic and familial AD. However, identifying common metabolic deficits in most AD patients is undoubtedly required before producing the mitochondrial function as a clinically useful biomarker.

Third, achieve tissue selectivity for the drug to reach mitochondria by penetrating the blood–brain barrier to minimize other side effects. Some proposed ways to attain this include selective activation of pro-drug by enzymes, combined delivery of more than one active compound targeting mitochondria that react with each other after reaching mitochondria, or radiotherapeutic approaches [255, 257, 258].

## Conclusion

Understanding mitochondrial function in AD is challenging due to the complex nature of the mitochondrial network and its interactions with other cellular components. Some therapeutic modalities have shown promising results in preclinical models, including antioxidants, CoQ10, melatonin, olesoxime, small peptide SS-31, lithium, vaccines, berberine, and pioglitazone. However, it is crucial to acknowledge that current animal and human cell models do not fully replicate AD or the complexity of the human brain. Furthermore, AD’s varied potential causes and progression paths add another layer of complexity to the research. Future work should prioritize understanding the progression of AD and the mitochondria-associated biomarkers at each stage. This knowledge can then be used to develop formulations targeting these biomarkers in mitochondria while optimizing tissue selectivity. Meanwhile, it is essential to optimize the use of current FDA-approved medications to manage the symptoms of AD, tailoring them to the specific needs of patients.

## Data Availability

No datasets were generated or analysed during the current study.

## References

[1] Alkon D, Sun MK, Thompson R (2021) Evidence of significant cognitive improvement over baseline in advanced Alzheimer’s disease (AD) patients: a regenerative therapeutic strategy. Alzheimers Dement 17(S9):e050013

[2] Kashif M, Sivaprakasam P, Vijendra P, Waseem M, Pandurangan AK (2023) A recent update on pathophysiology and therapeutic interventions of Alzheimer’s disease. Curr Pharm Des. 10.2174/0113816128264355231121064704 10.2174/0113816128264355231121064704 38038007

[3] Varadharajan A, Davis AD, Ghosh A, Jagtap T, Xavier A, Menon AJ et al (2023) Guidelines for pharmacotherapy in Alzheimer’s disease – a primer on FDA-approved drugs. J Neurosci Rural Pract 14(4):566–573 38059250 10.25259/JNRP_356_2023 PMC10696336

[4] Gettman L (2024) Lecanemab-irmb (Leqembi™) for treatment of Alzheimer’s disease. Sr Care Pharm 39(2):75–77 38263569 10.4140/TCP.n.2024.75

[5] Huang M, Bargues-Carot A, Riaz Z, Wickham H, Zenitsky G, Jin H et al (2022) Impact of environmental risk factors on mitochondrial dysfunction, neuroinflammation, protein misfolding, and oxidative stress in the etiopathogenesis of Parkinson’s disease. Int J Mol Sci 23(18):10808 36142718 10.3390/ijms231810808 PMC9505762

[6] Nabi SU, Khan A, Siddiqui EM, Rehman MU, Alshahrani S, Arafah A et al (2022) Mechanisms of mitochondrial malfunction in Alzheimer’s disease: new therapeutic hope. Oxid Med Cell Longev 2022:4759963 35607703 10.1155/2022/4759963 PMC9124149

[7] Joshi M, Joshi S, Khambete M, Degani M (2023) Role of calcium dysregulation in Alzheimer’s disease and its therapeutic implications. Chem Biol Drug Des 101(2):453–468 36373976 10.1111/cbdd.14175

[8] Mani S, Swargiary G, Singh M, Agarwal S, Dey A, Ojha S et al (2021) Mitochondrial defects: an emerging theranostic avenue towards Alzheimer’s associated dysregulations. Life Sci 15(285):119985 10.1016/j.lfs.2021.119985 34592237

[9] Fracassi A, Marcatti M, Zolochevska O, Tabor N, Woltjer R, Moreno S et al (2021) Oxidative damage and antioxidant response in frontal cortex of demented and nondemented individuals with Alzheimer’s neuropathology. J Neurosci Off J Soc Neurosci 41(3):538–554 10.1523/JNEUROSCI.0295-20.2020 PMC7821866 33239403

[10] Du H, Guo L, Yan SS (2012) Synaptic mitochondrial pathology in Alzheimer’s disease. Antioxid Redox Signal 16(12):1467–1475 21942330 10.1089/ars.2011.4277 PMC3329948

[11] Manczak M, Reddy PH (2012) Abnormal interaction between the mitochondrial fission protein Drp1 and hyperphosphorylated tau in Alzheimer’s disease neurons: implications for mitochondrial dysfunction and neuronal damage. Hum Mol Genet 21(11):2538–2547 22367970 10.1093/hmg/dds072 PMC3349426

[12] Tranah GJ, Nalls MA, Katzman SM, Yokoyama JS, Lam ET, Zhao Y et al (2012) Mitochondrial DNA sequence variation associated with dementia and cognitive function in the elderly. J Alzheimers Dis JAD 32(2):357–372 22785396 10.3233/JAD-2012-120466 PMC4156011

[13] Perez Ortiz JM, Swerdlow RH (2019) Mitochondrial dysfunction in Alzheimer’s disease: role in pathogenesis and novel therapeutic opportunities. Br J Pharmacol 176(18):3489–3507 30675901 10.1111/bph.14585 PMC6715612

[14] Hampel H, Hardy J, Blennow K, Chen C, Perry G, Kim SH et al (2021) The amyloid-β pathway in Alzheimer’s disease. Mol Psychiatry 26(10):5481–5503 34456336 10.1038/s41380-021-01249-0 PMC8758495

[15] Brucato FH, Benjamin DE (2020) Synaptic pruning in Alzheimer’s disease: role of the complement system. Glob J Med Res 29:1–20 10.34257/gjmrfvol20is6pg1 PMC7518506 32982106

[16] Paasila PJ, Aramideh JA, Sutherland GT, Graeber MB (2022) Synapses, microglia, and lipids in Alzheimer’s disease. Front Neurosci 12(15):778822 10.3389/fnins.2021.778822 PMC8789683 35095394

[17] Rather MA, Khan A, Alshahrani S, Rashid H, Qadri M, Rashid S et al (2021) Inflammation and Alzheimer’s disease: mechanisms and therapeutic implications by natural products. Oliveira SHP, editor. Mediators Inflamm 2021:1–21. 10.1155/2021/9982954 10.1155/2021/9982954 PMC8352708 34381308

[18] Ramachandran AK, Das S, Joseph A, Shenoy GG, Alex AT, Mudgal J (2021) Neurodegenerative pathways in Alzheimer’s disease: a review. Curr Neuropharmacol 19(5):679–692 32851951 10.2174/1570159X18666200807130637 PMC8573750

[19] Chen ZR, Huang JB, Yang SL, Hong FF (2022) Role of cholinergic signaling in Alzheimer’s disease. Mol Basel Switz 27(6):1816 10.3390/molecules27061816 PMC8949236 35335180

[20] Wong KY, Roy J, Fung ML, Heng BC, Zhang C, Lim LW (2020) Relationships between mitochondrial dysfunction and neurotransmission failure in Alzheimer’s disease. Aging Dis 11(5):1291–1316 33014538 10.14336/AD.2019.1125 PMC7505271

[21] Tyagi S, Thakur AK (2023) Neuropharmacological study on capsaicin in scopolamine-injected mice. Curr Alzheimer Res 20(9):660–676 38213170 10.2174/0115672050286225231230130613

[22] Aldossary AM, Tawfik EA, Alomary MN, Alsudir SA, Alfahad AJ, Alshehri AA et al (2022) Recent advances in mitochondrial diseases: from molecular insights to therapeutic perspectives. Saudi Pharm J SPJ Off Publ Saudi Pharm Soc 30(8):1065–1078 10.1016/j.jsps.2022.05.011 PMC9508646 36164575

[23] Onyango IG, Bennett JP, Stokin GB (2021) Mitochondrially-targeted therapeutic strategies for Alzheimer’s disease. Curr Alzheimer Res 18(10):753–771 34879805 10.2174/1567205018666211208125855 PMC9178515

[24] Wang W, Zhao F, Ma X, Perry G, Zhu X (2020) Mitochondria dysfunction in the pathogenesis of Alzheimer’s disease: recent advances. Mol Neurodegener 15(1):30 32471464 10.1186/s13024-020-00376-6 PMC7257174

[25] Bélanger M, Allaman I, Magistretti PJ (2011) Brain energy metabolism: focus on astrocyte-neuron metabolic cooperation. Cell Metab 14(6):724–738 22152301 10.1016/j.cmet.2011.08.016

[26] Zheng PP, Romme E, van der Spek PJ, Dirven CMF, Willemsen R, Kros JM (2010) Glut1/SLC2A1 is crucial for the development of the blood-brain barrier in vivo. Ann Neurol 68(6):835–844 21194153 10.1002/ana.22318

[27] Kapogiannis D, Mattson MP (2011) Disrupted energy metabolism and neuronal circuit dysfunction in cognitive impairment and Alzheimer’s disease. Lancet Neurol 10(2):187–198 21147038 10.1016/S1474-4422(10)70277-5 PMC3026092

[28] Crane PK, Walker R, Hubbard RA, Li G, Nathan DM, Zheng H et al (2013) Glucose levels and risk of dementia. N Engl J Med 369(6):540–548 23924004 10.1056/NEJMoa1215740 PMC3955123

[29] Croteau E, Castellano CA, Fortier M, Bocti C, Fulop T, Paquet N et al (2018) A cross-sectional comparison of brain glucose and ketone metabolism in cognitively healthy older adults, mild cognitive impairment and early Alzheimer’s disease. Exp Gerontol 1(107):18–26 10.1016/j.exger.2017.07.004 28709938

[30] Gordon BA, Blazey TM, Su Y, Hari-Raj A, Dincer A, Flores S et al (2018) Spatial patterns of neuroimaging biomarker change in individuals from families with autosomal dominant Alzheimer’s disease: a longitudinal study. Lancet Neurol 17(3):241–250 29397305 10.1016/S1474-4422(18)30028-0 PMC5816717

[31] Paranjpe MD, Chen X, Liu M, Paranjpe I, Leal JP, Wang R et al (2019) The effect of ApoE ε4 on longitudinal brain region-specific glucose metabolism in patients with mild cognitive impairment: a FDG-PET study. NeuroImage Clin 1(22):101795 10.1016/j.nicl.2019.101795 PMC6449776 30991617

[32] Mount DL, Ashley AV, Lah JJ, Levey AI, Goldstein FC (2009) Is ApoE ε4 Associated with cognitive functioning in african americans diagnosed with alzheimer disease? an exploratory study. South Med J 102(9):890. 10.1097/SMJ.0b013e3181b21b82 19668025 10.1097/SMJ.0b013e3181b21b82 PMC4406227

[33] Hendrie HC, Murrell J, Baiyewu O, Lane KA, Purnell C, Ogunniyi A et al (2014) APOE ε4 and the risk for Alzheimer disease and cognitive decline in African Americans and Yoruba. Int Psychogeriatr 26(6):977–985 24565289 10.1017/S1041610214000167 PMC4012422

[34] Mortimer JA, Snowdon DA, Markesbery WR (2009) The effect of APOE-ε4 on dementia is mediated by Alzheimer neuropathology. Alzheimer Dis Assoc Disord 23(2):152 19484916 10.1097/wad.0b013e318190a855 PMC2752689

[35] Mormino EC, Betensky RA, Hedden T, Schultz AP, Ward A, Huijbers W et al (2014) Amyloid and APOE ε4 interact to influence short-term decline in preclinical Alzheimer disease. Neurology 82(20):1760–1767 24748674 10.1212/WNL.0000000000000431 PMC4035706

[36] Mishra S, Blazey TM, Holtzman DM, Cruchaga C, Su Y, Morris JC et al (2018) Longitudinal brain imaging in preclinical Alzheimer disease: impact of APOE ε4 genotype. Brain 141(6):1828–1839 29672664 10.1093/brain/awy103 PMC5972633

[37] Reiman EM, Chen K, Alexander GE, Caselli RJ, Bandy D, Osborne D et al (2004) Functional brain abnormalities in young adults at genetic risk for late-onset Alzheimer’s dementia. Proc Natl Acad Sci101(1):284–289 10.1073/pnas.2635903100 PMC314177 14688411

[38] Chen Z, Zhong C (2013) Decoding Alzheimer’s disease from perturbed cerebral glucose metabolism: implications for diagnostic and therapeutic strategies. Prog Neurobiol 1(108):21–43 10.1016/j.pneurobio.2013.06.004 23850509

[39] Weise CM, Chen K, Chen Y, Kuang X, Savage CR, Reiman EM (2018) Left lateralized cerebral glucose metabolism declines in amyloid-β positive persons with mild cognitive impairment. NeuroImage Clin 1(20):286–296 10.1016/j.nicl.2018.07.016 PMC6084012 30101060

[40] Arbizu J, Festari C, Altomare D, Walker Z, Bouwman F, Rivolta J et al (2018) Clinical utility of FDG-PET for the clinical diagnosis in MCI. Eur J Nucl Med Mol Imaging 45(9):1497–1508 29704037 10.1007/s00259-018-4039-7

[41] Sörensen A, Blazhenets G, Rücker G, Schiller F, Meyer PT, Frings L (2019) Prognosis of conversion of mild cognitive impairment to Alzheimer’s dementia by voxel-wise Cox regression based on FDG PET data. NeuroImage Clin 1(21):101637 10.1016/j.nicl.2018.101637 PMC6411907 30553760

[42] McDade E, Wang G, Gordon BA, Hassenstab J, Benzinger TLS, Buckles V et al (2018) Longitudinal cognitive and biomarker changes in dominantly inherited Alzheimer disease. Neurology 91(14):e1295–e1306 30217935 10.1212/WNL.0000000000006277 PMC6177272

[43] Benzinger TLS, Blazey T, Jack CR, Koeppe RA, Su Y, Xiong C et al (2013) Regional variability of imaging biomarkers in autosomal dominant Alzheimer’s disease. Proc Natl Acad Sci 110(47):E4502–E4509 10.1073/pnas.1317918110 PMC3839740 24194552

[44] Altmann A, Ng B, Landau SM, Jagust WJ, Greicius MD, for the Alzheimer’s Disease Neuroimaging Initiative (2015) Regional brain hypometabolism is unrelated to regional amyloid plaque burden. Brain 138(12):3734–3746 10.1093/brain/awv278 PMC4806718 26419799

[45] Mastroeni D, Khdour OM, Delvaux E, Nolz J, Olsen G, Berchtold N et al (2017) Nuclear but not mitochondrial-encoded oxidative phosphorylation genes are altered in aging, mild cognitive impairment, and Alzheimer’s disease. Alzheimers Dement 13(5):510–519 27793643 10.1016/j.jalz.2016.09.003 PMC5967608

[46] Liang WS, Reiman EM, Valla J, Dunckley T, Beach TG, Grover A et al (2008) Alzheimer’s disease is associated with reduced expression of energy metabolism genes in posterior cingulate neurons. Proc Natl Acad Sci 105(11):4441–4416 10.1073/pnas.0709259105 PMC2393743 18332434

[47] Minoshima S, Giordani B, Berent S, Frey KA, Foster NL, Kuhl DE (1997) Metabolic reduction in the posterior cingulate cortex in very early Alzheimer’s disease. Ann Neurol 42(1):85–94 9225689 10.1002/ana.410420114

[48] Reiman EM, Caselli RJ, Yun LS, Chen K, Bandy D, Minoshima S et al (1996) Preclinical evidence of Alzheimer’s disease in persons homozygous for the ε4 allele for apolipoprotein E. N Engl J Med 334(12):752–758 8592548 10.1056/NEJM199603213341202

[49] Brooks WM, Lynch PJ, Ingle CC, Hatton A, Emson PC, Faull RLM et al (2007) Gene expression profiles of metabolic enzyme transcripts in Alzheimer’s disease. Brain Res 5(1127):127–135 10.1016/j.brainres.2006.09.106 17109828

[50] Burgoyne RD, Haynes LP (2012) Understanding the physiological roles of the neuronal calcium sensor proteins. Mol Brain 5(1):2 22269068 10.1186/1756-6606-5-2 PMC3271974

[51] Gleichmann M, Mattson MP (2011) Neuronal calcium homeostasis and dysregulation. Antioxid Redox Signal 14(7):1261–1273 20626318 10.1089/ars.2010.3386 PMC3048837

[52] Mochida S (2021) Neurotransmitter release site replenishment and presynaptic plasticity. Int J Mol Sci 22(1):327 10.3390/ijms22010327 PMC7794938 33396919

[53] Kannurpatti SS (2017) Mitochondrial calcium homeostasis: implications for neurovascular and neurometabolic coupling. J Cereb Blood Flow Metab 37(2):381–395 27879386 10.1177/0271678X16680637 PMC5381466

[54] Pedriali G, Rimessi A, Sbano L, Giorgi C, Wieckowski MR, Previati M, Pinton P (2017) Regulation of endoplasmic reticulum-mitochondria Ca2+ transfer and its importance for anti-cancer therapies. Front Oncol 7:286744. 10.3389/fonc.2017.00180 10.3389/fonc.2017.00180 PMC5583168 28913175

[55] Reddy PH (2013) Amyloid beta-induced glycogen synthase kinase 3β phosphorylated VDAC1 in Alzheimer’s disease: implications for synaptic dysfunction and neuronal damage. Biochim Biophys Acta BBA - Mol Basis Dis 1832(12):1913–1921 10.1016/j.bbadis.2013.06.012 PMC3825775 23816568

[56] Reddy PH (2013) Is the mitochondrial outermembrane protein VDAC1 therapeutic target for Alzheimer’s disease? Biochim Biophys Acta BBA - Mol Basis Dis 1832(1):67–75 10.1016/j.bbadis.2012.09.003 PMC3518645 22995655

[57] Finkel T, Menazza S, Holmström KM, Parks RJ, Liu J, Sun J et al (2015) The ins and outs of mitochondrial calcium. Circ Res 116(11):1810–1819 25999421 10.1161/CIRCRESAHA.116.305484 PMC6296495

[58] Shoshan-Barmatz V, De S (2017) Mitochondrial VDAC, the Na+/Ca2+ exchanger, and the Ca2+ uniporter in Ca2+ dynamics and signaling. In: Krebs J (eds) Membrane dynamics and calcium signaling. Advances in experimental medicine and biology, vol 981. Springer, Cham. 10.1007/978-3-319-55858-5_13 10.1007/978-3-319-55858-5_13 29594867

[59] Calvo-Rodriguez M, Hou SS, Snyder AC, Kharitonova EK, Russ AN, Das S et al (2020) Increased mitochondrial calcium levels associated with neuronal death in a mouse model of Alzheimer’s disease. Nat Commun 11(1):2146 32358564 10.1038/s41467-020-16074-2 PMC7195480

[60] Green KN (2009) Calcium in the initiation, progression and as an effector of Alzheimer’s disease pathology. J Cell Mol Med 13(9a):2787–2799 19650832 10.1111/j.1582-4934.2009.00861.x PMC4498936

[61] Tillement L, Lecanu L, Papadopoulos V (2011) Alzheimer’s disease: effects of β-amyloid on mitochondria. Mitochondrion 11(1):13–21 20817045 10.1016/j.mito.2010.08.009

[62] Berridge MJ (2014) Calcium regulation of neural rhythms, memory and Alzheimer’s disease. J Physiol 592(2):281–293 23753528 10.1113/jphysiol.2013.257527 PMC3922493

[63] Britti E, Delaspre F, Tamarit J, Ros J (2018) Mitochondrial calcium signalling and neurodegenerative diseases. Neuronal Signal 2(4):NS20180061 32714593 10.1042/NS20180061 PMC7373239

[64] John A, Reddy PH (2021) Synaptic basis of Alzheimer’s disease: focus on synaptic amyloid beta, P-tau and mitochondria. Ageing Res Rev 1(65):101208 10.1016/j.arr.2020.101208 PMC7770124 33157321

[65] Morton H, Kshirsagar S, Orlov E, Bunquin LE, Sawant N, Boleng L et al (2021) Defective mitophagy and synaptic degeneration in Alzheimer’s disease: focus on aging, mitochondria and synapse. Free Radic Biol Med 20(172):652–667 10.1016/j.freeradbiomed.2021.07.013 34246776

[66] Chen Y, Fu AKY, Ip NY (2019) Synaptic dysfunction in Alzheimer’s disease: mechanisms and therapeutic strategies. Pharmacol Ther 1(195):186–198 10.1016/j.pharmthera.2018.11.006 30439458

[67] Rajmohan R, Reddy PH (2017) Amyloid-beta and phosphorylated tau accumulations cause abnormalities at synapses of Alzheimer’s disease Neurons. J Alzheimers Dis 57(4):975–999 27567878 10.3233/JAD-160612 PMC5793225

[68] Calkins MJ, Reddy PH (2011) Amyloid beta impairs mitochondrial anterograde transport and degenerates synapses in Alzheimer’s disease neurons. Biochim Biophys Acta BBA - Mol Basis Dis 1812(4):507–513 10.1016/j.bbadis.2011.01.007 PMC3042500 21241801

[69] Tu S, Okamoto S ichi, Lipton SA, Xu H (2014) Oligomeric Aβ-induced synaptic dysfunction in Alzheimer’s disease. Mol Neurodegener 9(1):48 10.1186/1750-1326-9-48 PMC4237769 25394486

[70] Marsh J, Alifragis P (2018) Synaptic dysfunction in Alzheimer’s disease: the effects of amyloid beta on synaptic vesicle dynamics as a novel target for therapeutic intervention. Neural Regen Res 13(4):616 29722304 10.4103/1673-5374.230276 PMC5950662

[71] Zhou L, McInnes J, Wierda K, Holt M, Herrmann AG, Jackson RJ et al (2017) Tau association with synaptic vesicles causes presynaptic dysfunction. Nat Commun 8(1):15295 28492240 10.1038/ncomms15295 PMC5437271

[72] McInnes J, Wierda K, Snellinx A, Bounti L, Wang YC, Stancu IC et al (2018) Synaptogyrin-3 mediates presynaptic dysfunction induced by tau. Neuron 97(4):823-835.e8 29398363 10.1016/j.neuron.2018.01.022

[73] Dixit R, Ross JL, Goldman YE, Holzbaur ELF (2008) Differential regulation of dynein and kinesin motor proteins by tau. Science 319(5866):1086–1089 18202255 10.1126/science.1152993 PMC2866193

[74] Cheng Y, Bai F (2018) The association of Tau with mitochondrial dysfunction in Alzheimer's disease. Front Neurosci 12:320523. 10.3389/fnins.2018.00163 10.3389/fnins.2018.00163 PMC5874499 29623026

[75] Calafate S, Buist A, Miskiewicz K, Vijayan V, Daneels G, de Strooper B et al (2015) Synaptic contacts enhance cell-to-cell tau pathology propagation. Cell Rep 11(8):1176–1183 25981034 10.1016/j.celrep.2015.04.043

[76] Gowda P, Reddy PH, Kumar S (2022) Deregulated mitochondrial microRNAs in Alzheimer’s disease: focus on synapse and mitochondria. Ageing Res Rev 1(73):101529 10.1016/j.arr.2021.101529 PMC8692431 34813976

[77] Bell SM, Barnes K, De Marco M, Shaw PJ, Ferraiuolo L, Blackburn DJ et al (2021) Mitochondrial dysfunction in Alzheimer’s disease: a biomarker of the future? Biomedicines 9(1):63 33440662 10.3390/biomedicines9010063 PMC7827030

[78] Cai Q, Jeong YY (2020) Mitophagy in Alzheimer’s disease and other age-related neurodegenerative diseases. Cells 9(1):150 31936292 10.3390/cells9010150 PMC7017092

[79] Corsetti V, Florenzano F, Atlante A, Bobba A, Ciotti MT, Natale F et al (2015) NH2-truncated human tau induces deregulated mitophagy in neurons by aberrant recruitment of Parkin and UCHL-1: implications in Alzheimer’s disease. Hum Mol Genet 24(11):3058–3081 25687137 10.1093/hmg/ddv059

[80] Cummins N, Tweedie A, Zuryn S, Bertran-Gonzalez J, Götz J (2019) Disease-associated tau impairs mitophagy by inhibiting Parkin translocation to mitochondria. EMBO J 38(3):e99360 30538104 10.15252/embj.201899360 PMC6356067

[81] Hu Y, Li XC, Wang Z-H, Luo Y, Zhang X, Liu XP et al (2016) Tau accumulation impairs mitophagy via increasing mitochondrial membrane potential and reducing mitochondrial Parkin. Oncotarget 7(14):17356–68 26943044 10.18632/oncotarget.7861 PMC4951217

[82] Monteiro-Cardoso VF, Oliveira MM, Melo T, Domingues MRM, Moreira PI, Ferreiro E et al (2015) Cardiolipin profile changes are associated to the early synaptic mitochondrial dysfunction in Alzheimer’s disease. J Alzheimers Dis JAD 43(4):1375–1392 25182746 10.3233/JAD-141002

[83] Ye X, Sun X, Starovoytov V, Cai Q (2015) Parkin-mediated mitophagy in mutant hAPP neurons and Alzheimer’s disease patient brains. Hum Mol Genet 24(10):2938–2951 25678552 10.1093/hmg/ddv056 PMC4406302

[84] Cataldo AM, Peterhoff CM, Schmidt SD, Terio NB, Duff K, Beard M et al (2004) Presenilin mutations in familial Alzheimer disease and transgenic mouse models accelerate neuronal lysosomal pathology. J Neuropathol Exp Neurol 63(8):821–830 15330337 10.1093/jnen/63.8.821

[85] Ji ZS, Müllendorff K, Cheng IH, Miranda RD, Huang Y, Mahley RW (2006) Reactivity of apolipoprotein E4 and amyloid beta peptide: lysosomal stability and neurodegeneration. J Biol Chem 281(5):2683–2692 16298992 10.1074/jbc.M506646200

[86] Bernardi P (2013) The mitochondrial permeability transition pore: a mystery solved? Front Physiol 4:95 23675351 10.3389/fphys.2013.00095 PMC3650560

[87] Pérez MJ, Ponce DP, Aranguiz A, Behrens MI, Quintanilla RA (2018) Mitochondrial permeability transition pore contributes to mitochondrial dysfunction in fibroblasts of patients with sporadic Alzheimer’s disease. Redox Biol 19:290–300 30199818 10.1016/j.redox.2018.09.001 PMC6129674

[88] Sharma C, Kim S, Nam Y, Jung UJ, Kim SR (2021) Mitochondrial dysfunction as a driver of cognitive impairment in Alzheimer’s disease. Int J Mol Sci 22(9):4850 34063708 10.3390/ijms22094850 PMC8125007

[89] Supnet C, Bezprozvanny I (2010) The dysregulation of intracellular calcium in Alzheimer disease. Cell Calcium 47(2):183–189 20080301 10.1016/j.ceca.2009.12.014 PMC2834825

[90] Harada CN, Natelson Love MC, Triebel KL (2013) Normal cognitive aging. Clin Geriatr Med 29(4):737–752 24094294 10.1016/j.cger.2013.07.002 PMC4015335

[91] Qiu C, Kivipelto M, von Strauss E (2009) Epidemiology of Alzheimer’s disease: occurrence, determinants, and strategies toward intervention. Dialogues Clin Neurosci 11(2):111–128 19585947 10.31887/DCNS.2009.11.2/cqiu PMC3181909

[92] Savva GM, Wharton SB, Ince PG, Forster G, Matthews FE, Brayne C et al (2009) Age, neuropathology, and dementia. N Engl J Med 360(22):2302–2309 19474427 10.1056/NEJMoa0806142

[93] Payne BAI, Chinnery PF (2015) Mitochondrial dysfunction in aging: much progress but many unresolved questions. Biochim Biophys Acta 1847(11):1347–1353 26050973 10.1016/j.bbabio.2015.05.022 PMC4580208

[94] Harman D (1956) Aging: a theory based on free radical and radiation chemistry. J Gerontol 11(3):298–300 13332224 10.1093/geronj/11.3.298

[95] Finkel T, Holbrook NJ (2000) Oxidants, oxidative stress and the biology of ageing. Nature 408(6809):239–247 11089981 10.1038/35041687

[96] López-Otín C, Blasco MA, Partridge L, Serrano M, Kroemer G (2013) The hallmarks of aging. Cell 153(6):1194–1217 23746838 10.1016/j.cell.2013.05.039 PMC3836174

[97] Nisbet RM, Polanco JC, Ittner LM, Götz J (2015) Tau aggregation and its interplay with amyloid-β. Acta Neuropathol (Berl) 129(2):207–220 25492702 10.1007/s00401-014-1371-2 PMC4305093

[98] Meyer A, Laverny G, Bernardi L, Charles AL, Alsaleh G, Pottecher J et al (2018) Mitochondria: an organelle of bacterial origin controlling inflammation. Front Immunol 19(9):536 10.3389/fimmu.2018.00536 PMC5916961 29725325

[99] Wilkins HM, Carl SM, Greenlief ACS, Festoff BW, Swerdlow RH (2014) Bioenergetic dysfunction and inflammation in Alzheimer’s disease: a possible connection. Front Aging Neurosci 6:311 25426068 10.3389/fnagi.2014.00311 PMC4226164

[100] Bajwa E, Pointer CB, Klegeris A (2019) The role of mitochondrial damage-associated molecular patterns in chronic neuroinflammation. Mediators Inflamm 2019:4050796 31065234 10.1155/2019/4050796 PMC6466851

[101] Wilkins HM, Koppel SJ, Weidling IW, Roy N, Ryan LN, Stanford JA et al (2016) Extracellular mitochondria and mitochondrial components act as damage-associated molecular pattern molecules in the mouse brain. J Neuroimmune Pharmacol Off J Soc NeuroImmune Pharmacol 11(4):622–628 10.1007/s11481-016-9704-7 PMC5097888 27562848

[102] Guerreiro R, Wojtas A, Bras J, Carrasquillo M, Rogaeva E, Majounie E et al (2013) TREM2 variants in Alzheimer’s disease. N Engl J Med 368(2):117–127 23150934 10.1056/NEJMoa1211851 PMC3631573

[103] Wang Y, Cella M, Mallinson K, Ulrich JD, Young KL, Robinette ML et al (2015) TREM2 lipid sensing sustains the microglial response in an Alzheimer’s disease model. Cell 160(6):1061–1071 25728668 10.1016/j.cell.2015.01.049 PMC4477963

[104] Turnbull IR, Gilfillan S, Cella M, Aoshi T, Miller M, Piccio L et al (2006) Cutting edge: TREM-2 attenuates macrophage activation. J Immunol Baltim Md 1950 177(6):3520–4 10.4049/jimmunol.177.6.3520 16951310

[105] Jiang T, Zhang YD, Chen Q, Gao Q, Zhu XC, Zhou JS et al (2016) TREM2 modifies microglial phenotype and provides neuroprotection in P301S tau transgenic mice. Neuropharmacology 105:196–206 26802771 10.1016/j.neuropharm.2016.01.028

[106] Yan MH, Wang X, Zhu X (2013) Mitochondrial defects and oxidative stress in Alzheimer disease and Parkinson disease. Free Radic Biol Med 62:90–101 23200807 10.1016/j.freeradbiomed.2012.11.014 PMC3744189

[107] Wang J, Xiong S, Xie C, Markesbery WR, Lovell MA (2005) Increased oxidative damage in nuclear and mitochondrial DNA in Alzheimer’s disease. J Neurochem 93(4):953–962 15857398 10.1111/j.1471-4159.2005.03053.x

[108] Hirai K, Aliev G, Nunomura A, Fujioka H, Russell RL, Atwood CS et al (2001) Mitochondrial abnormalities in Alzheimer’s disease. J Neurosci Off J Soc Neurosci 21(9):3017–3023 10.1523/JNEUROSCI.21-09-03017.2001 PMC6762571 11312286

[109] Krishnan KJ, Ratnaike TE, De Gruyter HLM, Jaros E, Turnbull DM (2012) Mitochondrial DNA deletions cause the biochemical defect observed in Alzheimer’s disease. Neurobiol Aging 33(9):2210–2214 21925769 10.1016/j.neurobiolaging.2011.08.009

[110] Chen Y, Liu C, Parker WD, Chen H, Beach TG, Liu X et al (2016) Mitochondrial DNA rearrangement spectrum in brain tissue of Alzheimer’s disease: analysis of 13 cases. PLoS ONE 11(6):e0154582 27299301 10.1371/journal.pone.0154582 PMC4907522

[111] Mancuso M, Calsolaro V, Orsucci D, Carlesi C, Choub A, Piazza S et al (2009) Mitochondria, cognitive impairment, and Alzheimer’s disease. Int J Alzheimers Dis 6(2009):951548 10.4061/2009/951548 PMC2925259 20798880

[112] Blanch M, Mosquera JL, Ansoleaga B, Ferrer I, Barrachina M (2016) Altered mitochondrial DNA methylation pattern in Alzheimer disease-related pathology and in Parkinson disease. Am J Pathol 186(2):385–397 26776077 10.1016/j.ajpath.2015.10.004

[113] Stoccoro A, Siciliano G, Migliore L, Coppedè F (2017) Decreased methylation of the mitochondrial D-loop region in late-onset Alzheimer’s disease. J Alzheimers Dis JAD 59(2):559–564 28655136 10.3233/JAD-170139

[114] Oka T, Hikoso S, Yamaguchi O, Taneike M, Takeda T, Tamai T et al (2012) Mitochondrial DNA that escapes from autophagy causes inflammation and heart failure. Nature 485(7397):251–255 22535248 10.1038/nature10992 PMC3378041

[115] Simoncini C, Orsucci D, Caldarazzo Ienco E, Siciliano G, Bonuccelli U, Mancuso M (2015) Alzheimer’s pathogenesis and its link to the mitochondrion. Oxid Med Cell Longev 2015:803942 25973139 10.1155/2015/803942 PMC4417983

[116] Zhang L, Fang Y, Zhao X, Zheng Y, Ma Y, Li S et al (2021) miR-204 silencing reduces mitochondrial autophagy and ROS production in a murine AD model via the TRPML1-activated STAT3 pathway. Mol Ther Nucleic Acids 4(24):822–831 10.1016/j.omtn.2021.02.010 PMC8121631 34026326

[117] Sarkar S, Jun S, Rellick S, Quintana DD, Cavendish JZ, Simpkins JW (2016) Expression of microRNA-34a in Alzheimer’s disease brain targets genes linked to synaptic plasticity, energy metabolism, and resting state network activity. Brain Res 1(1646):139–151 10.1016/j.brainres.2016.05.026 PMC4975975 27235866

[118] Chen FZ, Zhao Y, Chen HZ (2019) MicroRNA-98 reduces amyloid β-protein production and improves oxidative stress and mitochondrial dysfunction through the Notch signaling pathway via HEY2 in Alzheimer’s disease mice. Int J Mol Med 43(1):91–102 30365070 10.3892/ijmm.2018.3957 PMC6257854

[119] Lang A, Grether-Beck S, Singh M, Kuck F, Jakob S, Kefalas A et al (2016) MicroRNA-15b regulates mitochondrial ROS production and the senescence-associated secretory phenotype through sirtuin 4/SIRT4. Aging 8(3):484–505 26959556 10.18632/aging.100905 PMC4833141

[120] Remenyi J, van den Bosch MWM, Palygin O, Mistry RB, McKenzie C, Macdonald A et al (2013) miR-132/212 knockout mice reveal roles for these miRNAs in regulating cortical synaptic transmission and plasticity. PLoS ONE 8(4):e62509 23658634 10.1371/journal.pone.0062509 PMC3637221

[121] Wingo TS, Yang J, Fan W, Min Canon S, Gerasimov ES, Lori A et al (2020) Brain microRNAs associated with late-life depressive symptoms are also associated with cognitive trajectory and dementia. NPJ Genomic Med 5:6 10.1038/s41525-019-0113-8 PMC7004995 32047652

[122] Di Rita A, Maiorino T, Bruqi K, Volpicelli F, Bellenchi GC, Strappazzon F (2020) miR-218 inhibits mitochondrial clearance by targeting PRKN E3 ubiquitin ligase. Int J Mol Sci 21(1):355 31948106 10.3390/ijms21010355 PMC6981953

[123] Chaudhuri AD, Choi DC, Kabaria S, Tran A, Junn E (2016) MicroRNA-7 regulates the function of mitochondrial permeability transition pore by targeting VDAC1 expression. J Biol Chem 291(12):6483–6493 26801612 10.1074/jbc.M115.691352 PMC4813563

[124] Li J, Donath S, Li Y, Qin D, Prabhakar BS, Li P (2010) miR-30 regulates mitochondrial fission through targeting p53 and the dynamin-related protein-1 pathway. PLOS Genet 6(1):e1000795 20062521 10.1371/journal.pgen.1000795 PMC2793031

[125] Goel P, Chakrabarti S, Goel K, Bhutani K, Chopra T, Bali S (2022) Neuronal cell death mechanisms in Alzheimer’s disease: an insight. Front Mol Neurosci 15:937133 36090249 10.3389/fnmol.2022.937133 PMC9454331

[126] Swerdlow RH (2007) Is aging part of Alzheimer’s disease, or is Alzheimer’s disease part of aging? Neurobiol Aging 28(10):1465–1480 16876913 10.1016/j.neurobiolaging.2006.06.021

[127] Blacker D, Tanzi RE (1998) The genetics of Alzheimer disease: current status and future prospects. Arch Neurol 55(3):294–296 9520001 10.1001/archneur.55.3.294

[128] Gaignard P, Liere P, Thérond P, Schumacher M, Slama A, Guennoun R (2017) Role of sex hormones on brain mitochondrial function, with special reference to aging and neurodegenerative diseases. Front Aging Neurosci 9:406 29270123 10.3389/fnagi.2017.00406 PMC5725410

[129] Guevara R, Gianotti M, Roca P, Oliver J (2011) Age and sex-related changes in rat brain mitochondrial function. Cell Physiol Biochem Int J Exp Cell Physiol Biochem Pharmacol 27(3–4):201–206 10.1159/000327945 21471708

[130] Lejri I, Grimm A, Eckert A (2018) Mitochondria, estrogen and female brain aging. Front Aging Neurosci 10:341477. 10.3389/fnagi.2018.00124 10.3389/fnagi.2018.00124 PMC5934418 29755342

[131] Zhao W, Hou Y, Song X, Wang L, Zhang F, Zhang H et al (2021) Estrogen deficiency induces mitochondrial damage prior to emergence of cognitive deficits in a postmenopausal mouse model. Front Aging Neurosci 13:713819 34335235 10.3389/fnagi.2021.713819 PMC8319728

[132] Gaignard P, Fréchou M, Schumacher M, Thérond P, Mattern C, Slama A et al (2016) Progesterone reduces brain mitochondrial dysfunction after transient focal ischemia in male and female mice. J Cereb Blood Flow Metab 36(3):562–568 26661198 10.1177/0271678X15610338 PMC4794096

[133] Yan W, Kang Y, Ji X, Li S, Li Y, Zhang G et al (2017) Testosterone upregulates the expression of mitochondrial ND1 and ND4 and alleviates the oxidative damage to the nigrostriatal dopaminergic system in orchiectomized rats. Oxid Med Cell Longev 2017:1202459 29138672 10.1155/2017/1202459 PMC5613679

[134] Parker WD, Parks J, Filley CM, Kleinschmidt-DeMasters BK (1994) Electron transport chain defects in Alzheimer’s disease brain. Neurology 44(6):1090–1096 8208407 10.1212/wnl.44.6.1090

[135] Cottrell DA, Blakely EL, Johnson MA, Ince PG, Turnbull DM (2001) Mitochondrial enzyme-deficient hippocampal neurons and choroidal cells in AD. Neurology 57(2):260–264 11468310 10.1212/wnl.57.2.260

[136] Maurer I, Zierz S, Möller H (2000) A selective defect of cytochrome c oxidase is present in brain of Alzheimer disease patients. Neurobiol Aging 21(3):455–462 10858595 10.1016/s0197-4580(00)00112-3

[137] Mutisya EM, Bowling AC, Beal MF (1994) Cortical cytochrome oxidase activity is reduced in Alzheimer’s disease. J Neurochem 63(6):2179–2184 7964738 10.1046/j.1471-4159.1994.63062179.x

[138] Mastrogiacoma F, Lindsay JG, Bettendorff L, Rice J, Kish SJ (1996) Brain protein and alpha-ketoglutarate dehydrogenase complex activity in Alzheimer’s disease. Ann Neurol 39(5):592–598 8619544 10.1002/ana.410390508

[139] Gibson GE, Park LC, Sheu KF, Blass JP, Calingasan NY (2000) The alpha-ketoglutarate dehydrogenase complex in neurodegeneration. Neurochem Int 36(2):97–112 10676873 10.1016/s0197-0186(99)00114-x

[140] Ko LW, Sheu KF, Thaler HT, Markesbery WR, Blass JP (2001) Selective loss of KGDHC-enriched neurons in Alzheimer temporal cortex: does mitochondrial variation contribute to selective vulnerability? J Mol Neurosci MN 17(3):361–369 11859932 10.1385/JMN:17:3:361

[141] Abudhaise H, Taanman JW, DeMuylder P, Fuller B, Davidson BR (2021) Mitochondrial respiratory chain and Krebs cycle enzyme function in human donor livers subjected to end-ischaemic hypothermic machine perfusion. PLoS ONE 16(10):e0257783 34710117 10.1371/journal.pone.0257783 PMC8553115

[142] Bubber P, Haroutunian V, Fisch G, Blass JP, Gibson GE (2005) Mitochondrial abnormalities in Alzheimer brain: mechanistic implications. Ann Neurol 57(5):695–703 15852400 10.1002/ana.20474

[143] Schulman MP, Richert DA (1957) Heme synthesis in vitamin B6 and pantothenic acid deficiencies. J Biol Chem 226(1):181–189 13428751

[144] Furuyama K, Sassa S (2000) Interaction between succinyl CoA synthetase and the heme-biosynthetic enzyme ALAS-E is disrupted in sideroblastic anemia. J Clin Invest 105(6):757–764 10727444 10.1172/JCI6816 PMC377455

[145] Atamna H, Frey WH (2007) Mechanisms of mitochondrial dysfunction and energy deficiency in Alzheimer’s disease. Mitochondrion 7(5):297–310 17625988 10.1016/j.mito.2007.06.001

[146] Atamna H, Newberry J, Erlitzki R, Schultz CS, Ames BN (2007) Biotin deficiency inhibits heme synthesis and impairs mitochondria in human lung fibroblasts. J Nutr 137(1):25–30 17182796 10.1093/jn/137.1.25

[147] Song Y, Zhu XY, Zhang XM, Xiong H (2022) Targeted mitochondrial epigenetics: a new direction in Alzheimer’s disease treatment. Int J Mol Sci 23(17):9703 36077101 10.3390/ijms23179703 PMC9456144

[148] Eckert GP, Eckert SH, Eckmann J, Hagl S, Muller WE, Friedland K (2020) Olesoxime improves cerebral mitochondrial dysfunction and enhances Aβ levels in preclinical models of Alzheimer’s disease. Exp Neurol 329:113286 32199815 10.1016/j.expneurol.2020.113286

[149] Grewal R, Reutzel M, Dilberger B, Hein H, Zotzel J, Marx S et al (2020) Purified oleocanthal and ligstroside protect against mitochondrial dysfunction in models of early Alzheimer’s disease and brain ageing. Exp Neurol 328:113248 32084452 10.1016/j.expneurol.2020.113248

[150] Tu JL, Chen WP, Cheng ZJ, Zhang G, Luo QH, Li M et al (2020) EGb761 ameliorates cell necroptosis by attenuating RIP1-mediated mitochondrial dysfunction and ROS production in both in vivo and in vitro models of Alzheimer’s disease. Brain Res 1(1736):146730 10.1016/j.brainres.2020.146730 32081533

[151] Zhang XY, Meng Y, Yan XJ, Liu S, Wang GQ, Cao YP (2021) Immunization with Aβ3-10-KLH vaccine improves cognitive function and ameliorates mitochondrial dysfunction and reduces Alzheimer’s disease-like pathology in Tg-APPswe/PSEN1dE9 mice. Brain Res Bull 174:31–40 34044034 10.1016/j.brainresbull.2021.05.019

[152] Lauretti E, Dincer O, Praticò D (2020) Glycogen synthase kinase-3 signaling in Alzheimer’s disease. Biochim Biophys Acta Mol Cell Res 1867(5):118664 32006534 10.1016/j.bbamcr.2020.118664 PMC7047718

[153] Dai DF, Chiao YA, Martin GM, Marcinek DJ, Basisty N, Quarles EK et al (2017) Mitochondrial-targeted catalase: extended longevity and the roles in various disease models. Prog Mol Biol Transl Sci 146:203–241 28253986 10.1016/bs.pmbts.2016.12.015

[154] McCormick B, Lowes DA, Colvin L, Torsney C, Galley HF (2016) MitoVitE, a mitochondria-targeted antioxidant, limits paclitaxel-induced oxidative stress and mitochondrial damage in vitro, and paclitaxel-induced mechanical hypersensitivity in a rat pain model. Br J Anaesth 117(5):659–666 27799181 10.1093/bja/aew309

[155] Arlt S, Müller-Thomsen T, Beisiegel U, Kontush A (2012) Effect of one-year vitamin C- and E-supplementation on cerebrospinal fluid oxidation parameters and clinical course in Alzheimer’s disease. Neurochem Res 37(12):2706–2714 22878647 10.1007/s11064-012-0860-8

[156] Dong R, Yang Q, Zhang Y, Li J, Zhang L, Zhao H (2018) Meta-analysis of vitamin C, vitamin E and β-carotene levels in the plasma of Alzheimer’s disease patients. Wei Sheng Yan Jiu 47(4):648–654 30081996

[157] Misrani A, Tabassum S, Yang L (2021) Mitochondrial dysfunction and oxidative stress in Alzheimer’s disease. Front Aging Neurosci 13:617588. 10.3389/fnagi.2021.617588 10.3389/fnagi.2021.617588 PMC7930231 33679375

[158] Mao P, Manczak M, Calkins MJ, Truong Q, Reddy TP, Reddy AP et al (2012) Mitochondria-targeted catalase reduces abnormal APP processing, amyloid β production and BACE1 in a mouse model of Alzheimer’s disease: implications for neuroprotection and lifespan extension. Hum Mol Genet 21(13):2973–2990 22492996 10.1093/hmg/dds128 PMC3373244

[159] Tardiolo G, Bramanti P, Mazzon E (2018) Overview on the effects of N-acetylcysteine in neurodegenerative diseases. Mol Basel Switz 23(12):3305 10.3390/molecules23123305 PMC6320789 30551603

[160] Spindler M, Beal MF, Henchcliffe C (2009) Coenzyme Q10 effects in neurodegenerative disease. Neuropsychiatr Dis Treat 5:597–610 19966907 10.2147/ndt.s5212 PMC2785862

[161] Jiménez-Jiménez FJ, Alonso-Navarro H, García-Martín E, Agúndez JAG (2023) Coenzyme Q10 and dementia: a systematic review. Antioxidants 12(2):533 36830090 10.3390/antiox12020533 PMC9952341

[162] Basile GA, Iannuzzo F, Xerra F, Genovese G, Pandolfo G, Cedro C et al (2023) Cognitive and mood effect of alpha-lipoic acid supplementation in a nonclinical elder sample: an open-label pilot study. Int J Environ Res Public Health 20(3):2358 36767724 10.3390/ijerph20032358 PMC9916195

[163] Fava A, Pirritano D, Plastino M, Cristiano D, Puccio G, Colica C et al (2013) The effect of lipoic acid therapy on cognitive functioning in patients with Alzheimer’s disease. J Neurodegener Dis 2013:454253 26316990 10.1155/2013/454253 PMC4437336

[164] Tripathi AK, Ray AK, Mishra SK, Bishen SM, Mishra H, Khurana A (2023) Molecular and therapeutic insights of alpha-lipoic acid as a potential molecule for disease prevention. Rev Bras Farmacogn Orgao Of Soc Bras Farmacogn 33(2):272–287 10.1007/s43450-023-00370-1 PMC9904877 36778891

[165] Chen C, Yang C, Wang J, Huang X, Yu H, Li S et al (2021) Melatonin ameliorates cognitive deficits through improving mitophagy in a mouse model of Alzheimer’s disease. J Pineal Res 71(4):e12774 34617321 10.1111/jpi.12774

[166] O’Reilly JA, Lynch M (2012) Rosiglitazone improves spatial memory and decreases insoluble Aβ(1–42) in APP/PS1 mice. J Neuroimmune Pharmacol Off J Soc NeuroImmune Pharmacol 7(1):140–144 10.1007/s11481-011-9282-7 21617889

[167] Risner ME, Saunders AM, Altman JFB, Ormandy GC, Craft S, Foley IM et al (2006) Efficacy of rosiglitazone in a genetically defined population with mild-to-moderate Alzheimer’s disease. Pharmacogenomics J 6(4):246–254 16446752 10.1038/sj.tpj.6500369

[168] Harrington C, Sawchak S, Chiang C, Davies J, Donovan C, Saunders AM et al (2011) Rosiglitazone does not improve cognition or global function when used as adjunctive therapy to AChE inhibitors in mild-to-moderate Alzheimer’s disease: two phase 3 studies. Curr Alzheimer Res 8(5):592–606 21592048 10.2174/156720511796391935

[169] Li Y, Duffy KB, Ottinger MA, Ray B, Bailey JA, Holloway HW et al (2010) GLP-1 receptor stimulation reduces amyloid-β peptide accumulation and cytotoxicity in cellular and animal models of Alzheimer’s disease. J Alzheimers Dis JAD 19(4):1205–1219 20308787 10.3233/JAD-2010-1314 PMC2948479

[170] An J, Zhou Y, Zhang M, Xie Y, Ke S, Liu L et al (2019) Exenatide alleviates mitochondrial dysfunction and cognitive impairment in the 5×FAD mouse model of Alzheimer’s disease. Behav Brain Res 16(370):111932 10.1016/j.bbr.2019.111932 31082410

[171] Xie Y, Zheng J, Li S, Li H, Zhou Y, Zheng W et al (2021) GLP-1 improves the neuronal supportive ability of astrocytes in Alzheimer’s disease by regulating mitochondrial dysfunction via the cAMP/PKA pathway. Biochem Pharmacol 188:114578 33895160 10.1016/j.bcp.2021.114578

[172] Liao W, Xu J, Li B, Ruan Y, Li T, Liu J (2021) Deciphering the roles of metformin in Alzheimer’s disease: a snapshot. Front Pharmacol 12:728315 35153733 10.3389/fphar.2021.728315 PMC8829062

[173] Vial G, Detaille D, Guigas B (2019) Role of mitochondria in the mechanism(s) of action of metformin. Front Endocrinol 10:294 10.3389/fendo.2019.00294 PMC6514102 31133988

[174] Chen S, Gan D, Lin S, Zhong Y, Chen M, Zou X et al (2022) Metformin in aging and aging-related diseases: clinical applications and relevant mechanisms. Theranostics 12(6):2722–2740 35401820 10.7150/thno.71360 PMC8965502

[175] Howell JJ, Hellberg K, Turner M, Talbott G, Kolar MJ, Ross DS et al (2017) Metformin inhibits hepatic mTORC1 signaling via dose-dependent mechanisms involving AMPK and the TSC complex. Cell Metab 25(2):463–471 28089566 10.1016/j.cmet.2016.12.009 PMC5299044

[176] Chen JL, Luo C, Pu D, Zhang GQ, Zhao YX, Sun Y et al (2019) Metformin attenuates diabetes-induced tau hyperphosphorylation in vitro and in vivo by enhancing autophagic clearance. Exp Neurol 311:44–56 30219731 10.1016/j.expneurol.2018.09.008

[177] GBD 2016 Dementia Collaborators (2019) Global, regional, and national burden of Alzheimer’s disease and other dementias, 1990–2016: a systematic analysis for the Global Burden of Disease Study 2016. Lancet Neurol 18(1):88–106 10.1016/S1474-4422(18)30403-4 PMC6291454 30497964

[178] Birks JS, Grimley EJ (2015) Rivastigmine for Alzheimer’s disease. Cochrane Database Syst Rev 4:CD001191 10.1002/14651858.CD001191.pub3 25858345

[179] Birks JS, Harvey RJ (2018) Donepezil for dementia due to Alzheimer’s disease. Cochrane Database Syst Rev 6(6):CD001190 12917900 10.1002/14651858.CD001190

[180] Bartus RT (2000) On neurodegenerative diseases, models, and treatment strategies: lessons learned and lessons forgotten a generation following the cholinergic hypothesis. Exp Neurol 163(2):495–529 10833325 10.1006/exnr.2000.7397

[181] Jelic V, Winblad B (2016) Alzheimer disease. Donepezil and nursing home placement–benefits and costs. Nat Rev Neurol 12(1):11–13 26714658 10.1038/nrneurol.2015.237

[182] Winblad B, Black SE, Homma A, Schwam EM, Moline M, Xu Y et al (2009) Donepezil treatment in severe Alzheimer’s disease: a pooled analysis of three clinical trials. Curr Med Res Opin 25(11):2577–2587 19735164 10.1185/03007990903236731

[183] Marder K (2006) Donepezil in patients with severe Alzheimer’s disease: double-blind parallel-group, placebo controlled study. Curr Neurol Neurosci Rep 6(5):363–364 10.1007/s11910-996-0015-x 16928344

[184] Schmidt R, Hofer E, Bouwman FH, Buerger K, Cordonnier C, Fladby T et al (2015) EFNS-ENS/EAN Guideline on concomitant use of cholinesterase inhibitors and memantine in moderate to severe Alzheimer’s disease. Eur J Neurol 22(6):889–898 25808982 10.1111/ene.12707

[185] Tariot PN, Farlow MR, Grossberg GT, Graham SM, McDonald S, Gergel I et al (2004) Memantine treatment in patients with moderate to severe Alzheimer disease already receiving donepezil: a randomized controlled trial. JAMA 291(3):317–324 14734594 10.1001/jama.291.3.317

[186] Bakchine S, Loft H (2007) Memantine treatment in patients with mild to moderate Alzheimer’s disease: results of a randomised, double-blind, placebo-controlled 6-month study. J Alzheimers Dis JAD 11(4):471–479 17656827 10.3233/jad-2007-11409

[187] Howard R, McShane R, Lindesay J, Ritchie C, Baldwin A, Barber R et al (2012) Donepezil and memantine for moderate-to-severe Alzheimer’s disease. N Engl J Med 366(10):893–903 22397651 10.1056/NEJMoa1106668

[188] Grossberg GT, Manes F, Allegri RF, Gutiérrez-Robledo LM, Gloger S, Xie L et al (2013) The safety, tolerability, and efficacy of once-daily memantine (28 mg): a multinational, randomized, double-blind, placebo-controlled trial in patients with moderate-to-severe Alzheimer’s disease taking cholinesterase inhibitors. CNS Drugs 27(6):469–478 23733403 10.1007/s40263-013-0077-7 PMC3680656

[189] Religa D, Fereshtehnejad SM, Cermakova P, Edlund AK, Garcia-Ptacek S, Granqvist N et al (2015) SveDem, the Swedish Dementia Registry - a tool for improving the quality of diagnostics, treatment and care of dementia patients in clinical practice. PLoS ONE 10(2):e0116538 25695768 10.1371/journal.pone.0116538 PMC4335024

[190] Mesulam M (2004) The cholinergic lesion of Alzheimer’s disease: pivotal factor or side show? Learn Mem Cold Spring Harb N 11(1):43–49 10.1101/lm.69204 14747516

[191] Turnbull MT, Boskovic Z, Coulson EJ (2018) Acute down-regulation of BDNF signaling does not replicate exacerbated amyloid-β levels and cognitive impairment induced by cholinergic basal forebrain lesion. Front Mol Neurosci 11:51 29520217 10.3389/fnmol.2018.00051 PMC5827359

[192] Labrador-Espinosa MA, Silva-Rodríguez J, Reina-Castillo MI, Mir P, Grothe MJ (2023) Basal forebrain atrophy, cortical thinning, and amyloid-β status in Parkinson’s disease-related cognitive decline. Mov Disord Off J Mov Disord Soc 38(10):1871–1880 10.1002/mds.29564 37492892

[193] Li DD, Zhang YH, Zhang W, Zhao P (2019) Meta-analysis of randomized controlled trials on the efficacy and safety of donepezil, galantamine, rivastigmine, and memantine for the treatment of Alzheimer’s disease. Front Neurosci 13:472 31156366 10.3389/fnins.2019.00472 PMC6529534

[194] Farlow M, Anand R, Messina J, Hartman R, Veach J (2000) A 52-week study of the efficacy of rivastigmine in patients with mild to moderately severe Alzheimer’s disease. Eur Neurol 44(4):236–241 11096224 10.1159/000008243

[195] Winblad B, Engedal K, Soininen H, Verhey F, Waldemar G, Wimo A et al (2001) A 1-year, randomized, placebo-controlled study of donepezil in patients with mild to moderate AD. Neurology 57(3):489–495 11502918 10.1212/wnl.57.3.489

[196] Mohs RC, Doody RS, Morris JC, Ieni JR, Rogers SL, Perdomo CA et al (2001) A 1-year, placebo-controlled preservation of function survival study of donepezil in AD patients. Neurology 57(3):481–488 11502917 10.1212/wnl.57.3.481

[197] Courtney C, Farrell D, Gray R, Hills R, Lynch L, Sellwood E et al (2004) Long-term donepezil treatment in 565 patients with Alzheimer’s disease (AD2000): randomised double-blind trial. Lancet Lond Engl 363(9427):2105–2115 10.1016/S0140-6736(04)16499-4 15220031

[198] Karaman Y, Erdoğan F, Köseoğlu E, Turan T, Ersoy AO (2005) A 12-month study of the efficacy of rivastigmine in patients with advanced moderate Alzheimer’s disease. Dement Geriatr Cogn Disord 19(1):51–56 15383747 10.1159/000080972

[199] Wattmo C, Londos E, Minthon L (2015) Longitudinal associations between survival in Alzheimer’s disease and cholinesterase inhibitor use, progression, and community-based services. Dement Geriatr Cogn Disord 40(5–6):297–310 26335053 10.1159/000437050

[200] Vellas B, Hausner L, Frölich L, Cantet C, Gardette V, Reynish E et al (2012) Progression of Alzheimer disease in Europe: data from the European ICTUS study. Curr Alzheimer Res 9(8):902–912 22742853 10.2174/156720512803251066

[201] Wallin AK, Andreasen N, Eriksson S, Båtsman S, Nasman B, Ekdahl A et al (2007) Donepezil in Alzheimer’s disease: what to expect after 3 years of treatment in a routine clinical setting. Dement Geriatr Cogn Disord 23(3):150–160 17312368 10.1159/000098052

[202] Wattmo C, Londos E, Minthon L (2018) Short-term response to cholinesterase inhibitors in Alzheimer’s disease delays time to nursing home placement. Curr Alzheimer Res 15(10):905–916 29732972 10.2174/1567205015666180507105326 PMC6174634

[203] Xu H, Garcia-Ptacek S, Jönsson L, Wimo A, Nordström P, Eriksdotter M (2021) Long-term effects of cholinesterase inhibitors on cognitive decline and mortality. Neurology 96(17):e2220–e2230 33741639 10.1212/WNL.0000000000011832 PMC8166426

[204] Wattmo C, Londos E, Minthon L (2014) Response to cholinesterase inhibitors affects lifespan in Alzheimer’s disease. BMC Neurol 10(14):173 10.1186/s12883-014-0173-4 PMC4172846 25213579

[205] Tan ECK, Johnell K, Garcia-Ptacek S, Haaksma ML, Fastbom J, Bell JS et al (2018) Acetylcholinesterase inhibitors and risk of stroke and death in people with dementia. Alzheimers Dement J Alzheimers Assoc 14(7):944–951 10.1016/j.jalz.2018.02.011 29706487

[206] Nordström P, Religa D, Wimo A, Winblad B, Eriksdotter M (2013) The use of cholinesterase inhibitors and the risk of myocardial infarction and death: a nationwide cohort study in subjects with Alzheimer’s disease. Eur Heart J 34(33):2585–2591 23735859 10.1093/eurheartj/eht182

[207] Lin YT, Wu PH, Chen CS, Yang YH, Yang YH (2016) Association between acetylcholinesterase inhibitors and risk of stroke in patients with dementia. Sci Rep 5(6):29266 10.1038/srep29266 PMC4932523 27377212

[208] Corona C, Frazzini V, Silvestri E, Lattanzio R, Sorda RL, Piantelli M et al (2011) Effects of dietary supplementation of carnosine on mitochondrial dysfunction, amyloid pathology, and cognitive deficits in 3xTg-AD mice. PLoS ONE 6(3):e17971 21423579 10.1371/journal.pone.0017971 PMC3058055

[209] Billings LM, Oddo S, Green KN, McGaugh JL, LaFerla FM (2005) Intraneuronal Abeta causes the onset of early Alzheimer’s disease-related cognitive deficits in transgenic mice. Neuron 45(5):675–688 15748844 10.1016/j.neuron.2005.01.040

[210] Shekhar S, Yadav Y, Singh AP, Pradhan R, Desai GR, Dey AB et al (2018) Neuroprotection by ethanolic extract of Syzygium aromaticum in Alzheimer’s disease like pathology via maintaining oxidative balance through SIRT1 pathway. Exp Gerontol 110:277–283 29959974 10.1016/j.exger.2018.06.026

[211] Halder S, Mehta AK, Kar R, Mustafa M, Mediratta PK, Sharma KK (2011) Clove oil reverses learning and memory deficits in scopolamine-treated mice. Planta Med 77(08):830–834 21157682 10.1055/s-0030-1250605

[212] Durairajan SSK, Huang YY, Yuen PY, Chen LL, Kwok KY, Liu LF et al (2014) Effects of Huanglian-Jie-Du-Tang and its modified formula on the modulation of amyloid-β precursor protein processing in Alzheimer’s disease models. PLoS ONE 9(3):e92954 24671102 10.1371/journal.pone.0092954 PMC3966845

[213] Lu DY, Tang CH, Chen YH, Wei IH (2010) Berberine suppresses neuroinflammatory responses through AMP-activated protein kinase activation in BV-2 microglia. J Cell Biochem 110(3):697–705 20512929 10.1002/jcb.22580

[214] Wong LR, Tan EA, Lim MEJ, Shen W, Lian XL, Wang Y et al (2021) Functional effects of berberine in modulating mitochondrial dysfunction and inflammatory response in the respective amyloidogenic cells and activated microglial cells - in vitro models simulating Alzheimer’s disease pathology. Life Sci 1(282):119824 10.1016/j.lfs.2021.119824 34265361

[215] Stockburger C, Gold VAM, Pallas T, Kolesova N, Miano D, Leuner K et al (2014) A cell model for the initial phase of sporadic Alzheimer’s disease. J Alzheimers Dis JAD 42(2):395–411 24898661 10.3233/JAD-140381

[216] Gower AJ, Lamberty Y (1993) The aged mouse as a model of cognitive decline with special emphasis on studies in NMRI mice. Behav Brain Res 57(2):163–173 8117421 10.1016/0166-4328(93)90132-a

[217] Batarseh YS, Mohamed LA, Al Rihani SB, Mousa YM, Siddique AB, El Sayed KA et al (2017) Oleocanthal ameliorates amyloid-β oligomers’ toxicity on astrocytes and neuronal cells: in vitro studies. Neuroscience 3(352):204–215 10.1016/j.neuroscience.2017.03.059 PMC5504696 28392295

[218] Müller WE, Eckert A, Eckert GP, Fink H, Friedland K, Gauthier S et al (2019) Therapeutic efficacy of the Ginkgo special extract EGb761® within the framework of the mitochondrial cascade hypothesis of Alzheimer’s disease. World J Biol Psychiatry Off J World Fed Soc Biol Psychiatry 20(3):173–189 10.1080/15622975.2017.1308552 28460580

[219] Tian X, Zhang L, Wang J, Dai J, Shen S, Yang L et al (2013) The protective effect of hyperbaric oxygen and Ginkgo biloba extract on Aβ25-35-induced oxidative stress and neuronal apoptosis in rats. Behav Brain Res 1(242):1–8 10.1016/j.bbr.2012.12.026 23266522

[220] Thancharoen O, Limwattananon C, Waleekhachonloet O, Rattanachotphanit T, Limwattananon P, Limpawattana P (2019) Ginkgo biloba extract (EGb761), cholinesterase inhibitors, and memantine for the treatment of mild-to-moderate Alzheimer’s disease: a network meta-analysis. Drugs Aging 36(5):435–452 30937879 10.1007/s40266-019-00648-x

[221] Rabinovici GD, Gatsonis C, Apgar C, Chaudhary K, Gareen I, Hanna L et al (2019) Association of amyloid positron emission tomography with subsequent change in clinical management among medicare beneficiaries with mild cognitive impairment or dementia. JAMA 321(13):1286–1294 30938796 10.1001/jama.2019.2000 PMC6450276

[222] Ortiz A, Sansinenea E (2023) Phenylpropanoid derivatives and their role in plants’ health and as antimicrobials. Curr Microbiol 80(12):380 37864088 10.1007/s00284-023-03502-x

[223] Kolaj I, Imindu Liyanage S, Weaver DF (2018) Phenylpropanoids and Alzheimer’s disease: a potential therapeutic platform. Neurochem Int 1(120):99–111 10.1016/j.neuint.2018.08.001 30098379

[224] Zhang XW, Chen JY, Ouyang D, Lu JH (2020) Quercetin in animal models of Alzheimer’s disease: a systematic review of preclinical studies. Int J Mol Sci 21(2):493 31941000 10.3390/ijms21020493 PMC7014205

[225] Ho CL, Kao NJ, Lin CI, Cross TWL, Lin SH (2022) Quercetin increases mitochondrial biogenesis and reduces free radicals in neuronal SH-SY5Y cells. Nutrients 14(16):3310 36014814 10.3390/nu14163310 PMC9414536

[226] Paula PC, Angelica Maria SG, Luis CH, Gloria Patricia CG (2019) Preventive effect of quercetin in a triple transgenic Alzheimer’s disease mice model. Mol Basel Switz 24(12):2287 10.3390/molecules24122287 PMC6630340 31226738

[227] Tong-un T, Wannanon P, Wattanathorn J, Phachonpai W (2010) Cognitive-enhancing and antioxidant activities of quercetin liposomes in animal model of Alzheimer’s disease. OnLine J Biol Sci 10(2):84–91

[228] Brondino N, Re S, Boldrini A, Cuccomarino A, Lanati N, Barale F et al (2014) Curcumin as a therapeutic agent in dementia: a mini systematic review of human studies. ScientificWorldJournal 2014:174282 24578620 10.1155/2014/174282 PMC3919104

[229] Goozee KG, Shah TM, Sohrabi HR, Rainey-Smith SR, Brown B, Verdile G et al (2016) Examining the potential clinical value of curcumin in the prevention and diagnosis of Alzheimer’s disease. Br J Nutr 115(3):449–465 26652155 10.1017/S0007114515004687

[230] Berry A, Collacchi B, Masella R, Varì R, Cirulli F (2021) Curcuma longa, the “golden spice” to counteract neuroinflammaging and cognitive decline—what have we learned and what needs to be done. Nutrients 13(5):1519 33946356 10.3390/nu13051519 PMC8145550

[231] Chen M, Du ZY, Zheng X, Li DL, Zhou RP, Zhang K (2018) Use of curcumin in diagnosis, prevention, and treatment of Alzheimer’s disease. Neural Regen Res 13(4):742–752 29722330 10.4103/1673-5374.230303 PMC5950688

[232] Liu H, Li Z, Qiu D, Gu Q, Lei Q, Mao L (2010) The inhibitory effects of different curcuminoids on β-amyloid protein, β-amyloid precursor protein and β-site amyloid precursor protein cleaving enzyme 1 in swAPP HEK293 cells. Neurosci Lett 485(2):83–88 20727383 10.1016/j.neulet.2010.08.035

[233] Valera E, Dargusch R, Maher PA, Schubert D (2013) Modulation of 5-lipoxygenase in proteotoxicity and Alzheimer’s disease. J Neurosci Off J Soc Neurosci 33(25):10512–10525 10.1523/JNEUROSCI.5183-12.2013 PMC3685841 23785163

[234] Shabbir U, Rubab M, Tyagi A, Oh DH (2021) Curcumin and its derivatives as theranostic agents in Alzheimer’s disease: the implication of nanotechnology. Int J Mol Sci 22(1):196 10.3390/ijms22010196 PMC7795367 33375513

[235] Ding L, Meng Y, Zhang HY, Yin WC, Yan Y, Cao YP (2017) Prophylactic active immunization with a novel epitope vaccine improves cognitive ability by decreasing amyloid plaques and neuroinflammation in APP/PS1 transgenic mice. Neurosci Res 119:7–14 28111220 10.1016/j.neures.2017.01.003

[236] Li B, Liang F, Ding X, Yan Q, Zhao Y, Zhang X et al (2019) Interval and continuous exercise overcome memory deficits related to β-Amyloid accumulation through modulating mitochondrial dynamics. Behav Brain Res 376:112171 31445975 10.1016/j.bbr.2019.112171

[237] Sutherland RJ, McDonald RJ (1990) Hippocampus, amygdala, and memory deficits in rats. Behav Brain Res 37(1):57–79 2310495 10.1016/0166-4328(90)90072-m

[238] Akhtar A, Dhaliwal J, Sah SP (2021) 7,8-Dihydroxyflavone improves cognitive functions in ICV-STZ rat model of sporadic Alzheimer’s disease by reversing oxidative stress, mitochondrial dysfunction, and insulin resistance. Psychopharmacology 238(7):1991–2009 33774703 10.1007/s00213-021-05826-7

[239] Talboom JS, Velazquez R, Oddo S (2015) The mammalian target of rapamycin at the crossroad between cognitive aging and Alzheimer’s disease. NPJ Aging Mech Dis 1:15008 28721257 10.1038/npjamd.2015.8 PMC5514987

[240] Siegmund SE, Yang H, Sharma R, Javors M, Skinner O, Mootha V et al (2017) Low-dose rapamycin extends lifespan in a mouse model of mtDNA depletion syndrome. Hum Mol Genet 26(23):4588–4605 28973153 10.1093/hmg/ddx341 PMC5886265

[241] Koushki M, Amiri-Dashatan N, Ahmadi N, Abbaszadeh H, Rezaei-Tavirani M (2018) Resveratrol: a miraculous natural compound for diseases treatment. Food Sci Nutr 6(8):2473–2490 30510749 10.1002/fsn3.855 PMC6261232

[242] Zhou DD, Luo M, Huang SY, Saimaiti A, Shang A, Gan RY et al (2021) Effects and mechanisms of resveratrol on aging and age-related diseases. Oxid Med Cell Longev 2021:9932218 34336123 10.1155/2021/9932218 PMC8289612

[243] Sousa JCE, Santana ACF, MagalhÃes GJP (2020) Resveratrol in Alzheimer’s disease: a review of pathophysiology and therapeutic potential. Arq Neuropsiquiatr 78(8):501–511 32520230 10.1590/0004-282X20200010

[244] Ma X, Sun Z, Liu Y, Jia Y, Zhang B, Zhang J (2013) Resveratrol improves cognition and reduces oxidative stress in rats with vascular dementia. Neural Regen Res 8(22):2050–2059 25206513 10.3969/j.issn.1673-5374.2013.22.004 PMC4146064

[245] Ferreira AFF, Binda KH, Singulani MP, Pereira CPM, Ferrari GD, Alberici LC et al (2020) Physical exercise protects against mitochondria alterations in the 6-hidroxydopamine rat model of Parkinson’s disease. Behav Brain Res 1(387):112607 10.1016/j.bbr.2020.112607 32199987

[246] Singulani MP, De Paula VJR, Forlenza OV (2021) Mitochondrial dysfunction in Alzheimer’s disease: therapeutic implications of lithium. Neurosci Lett 24(760):136078 10.1016/j.neulet.2021.136078 34161823

[247] Forlenza OV, Diniz BS, Radanovic M, Santos FS, Talib LL, Gattaz WF (2011) Disease-modifying properties of long-term lithium treatment for amnestic mild cognitive impairment: randomised controlled trial. Br J Psychiatry J Ment Sci 198(5):351–356 10.1192/bjp.bp.110.080044 21525519

[248] De-Paula VJ, Gattaz WF, Forlenza OV (2016) Long-term lithium treatment increases intracellular and extracellular brain-derived neurotrophic factor (BDNF) in cortical and hippocampal neurons at subtherapeutic concentrations. Bipolar Disord 18(8):692–695 27882645 10.1111/bdi.12449

[249] Undi RB, Gutti U, Gutti RK (2017) LiCl regulates mitochondrial biogenesis during megakaryocyte development. J Trace Elem Med Biol Organ Soc Miner Trace Elem GMS 39:193–201 10.1016/j.jtemb.2016.10.003 27908414

[250] Ding XW, Robinson M, Li R, Aldhowayan H, Geetha T, Babu JR (2021) Mitochondrial dysfunction and beneficial effects of mitochondria-targeted small peptide SS-31 in Diabetes Mellitus and Alzheimer’s disease. Pharmacol Res 171:105783 34302976 10.1016/j.phrs.2021.105783

[251] Szeto HH (2006) Mitochondria-targeted peptide antioxidants: novel neuroprotective agents. AAPS J 8(3):E521–E531 17025271 10.1208/aapsj080362 PMC2761060

[252] Selkoe DJ, Yamazaki T, Citron M, Podlisny MB, Koo EH, Teplow DB et al (1996) The role of APP processing and trafficking pathways in the formation of amyloid beta-protein. Ann N Y Acad Sci 17(777):57–64 10.1111/j.1749-6632.1996.tb34401.x 8624127

[253] Lampinen R, Belaya I, Boccuni I, Kanninen TMKM, Lampinen R, Belaya I et al (2017) Mitochondrial function in Alzheimer’s disease: focus on astrocytes. In: Astrocyte - physiology and pathology [Internet]. IntechOpen.[cited 2024 May 5]. Available from: https://www.intechopen.com/chapters/58306

[254] Steele HE, Horvath R, Lyon JJ, Chinnery PF (2017) Monitoring clinical progression with mitochondrial disease biomarkers. Brain 140(10):2530–2540 28969370 10.1093/brain/awx168 PMC5841218

[255] Murphy MP, Hartley RC (2018) Mitochondria as a therapeutic target for common pathologies. Nat Rev Drug Discov 17(12):865–886 30393373 10.1038/nrd.2018.174

[256] Wojsiat J, Laskowska-Kaszub K, Mietelska-Porowska A, Wojda U (2017) Search for Alzheimer’s disease biomarkers in blood cells: hypotheses-driven approach. Biomark Med 11(10):917–931 28976776 10.2217/bmm-2017-0041

[257] Chalmers S, Caldwell ST, Quin C, Prime TA, James AM, Cairns AG et al (2012) Selective uncoupling of individual mitochondria within a cell using a mitochondria-targeted photoactivated protonophore. J Am Chem Soc 134(2):758–761 22239373 10.1021/ja2077922 PMC3260739

[258] Logan A, Pell VR, Shaffer KJ, Evans C, Stanley NJ, Robb EL et al (2016) Assessing the mitochondrial membrane potential in cells and in vivo using targeted click chemistry and mass spectrometry. Cell Metab 23(2):379–385 26712463 10.1016/j.cmet.2015.11.014 PMC4752821

